# Nuclear import of Mas-related G protein-coupled receptor member D induces pathological cardiac remodeling

**DOI:** 10.1186/s12964-023-01168-3

**Published:** 2023-07-24

**Authors:** Kun Zhao, Dongxu Hua, Chuanxi Yang, Xiaoguang Wu, Yukang Mao, Yanhui Sheng, Wei Sun, Yong Li, Xiangqing Kong, Peng Li

**Affiliations:** 1 Department of Cardiology, the First Affiliated Hospital of Nanjing Medical University, 300 Guangzhou Road, Nanjing, 210029 Jiangsu China; 2 Department of Cardiology, Yangpu Hospital, Tongji University School of Medicine, Shanghai, China; 3 Department of Cardiology, The Affiliated Suzhou Hospital of Nanjing Medical University, Suzhou Municipal Hospital, Gusu School, Nanjing Medical University, Suzhou, Jiangsu China

**Keywords:** Cardiac remodeling, Alamandine, Mas-related G protein-coupled receptor, Member D, Nuclear import, Gα_i_ subunit

## Abstract

Alamandine (Ala), a ligand of Mas-related G protein-coupled receptor, member D (MrgD), alleviates angiotensin II (AngII)-induced cardiac hypertrophy. However, the specific physiological and pathological role of MrgD is not yet elucidated. Here, we found that MrgD expression increased under various pathological conditions. Then, MrgD knockdown prevented AngII-induced cardiac hypertrophy and fibrosis via inactivating Gα_i_-mediacted downstream signaling pathways, including the phosphorylation of p38 (p-P38), while MrgD overexpression induced pathological cardiac remodeling. Next, Ala, like silencing MrgD, exerted its cardioprotective effects by inhibiting Ang II-induced nuclear import of MrgD. MrgD interacted with p-P38 and promoted its entry into the nucleus under Ang II stimulation. Our results indicated that Ala was a blocking ligand of MrgD that inhibited downstream signaling pathway, which unveiled the promising cardioprotective effect of silencing MrgD expression on alleviating cardiac remodeling.

Video Abstract

**Supplementary Information:**

The online version contains supplementary material available at 10.1186/s12964-023-01168-3.

## Background

Cardiac fibrosis and hypertrophy are the major hallmarks of cardiac remodeling that is involved in the pathophysiological process of heart diseases [1, 2]. However, they could disturb the function and structure of the myocardium [3, 4].

Previous studies have demonstrated that the renin–angiotensin–aldosterone system (RAAS) plays a vital pathophysiological role in the development of cardiovascular diseases (CVDs) [5, 6]. In addition to the classical renin/angiotensin-converting enzyme [7]/angiotensin (Ang) II/angiotensin type 1 receptor (AT1R) axis, several new biological compounds have been reported to exert a counter-regulatory effect against angiotensin II (Ang II)-induced fibrogenesis, especially Ang-(1–7)/Mas receptor and alamandine (Ala)/Mas-related G protein-coupled receptor (GPCR), member D (MrgD) axis [8, 9].

Ang-(1–7) has been identified to selectively protect against AngII-induced cardiac hypertrophy and fibrosis in hypertension [7, 10, 11]. Due to a similar molecular structure, Ala, which could be generated from Ang-(1–7) decarboxylation, has been considered to have biological properties similar to that of Ang-(1–7) [12, 13]. Moreover, our previous study demonstrated that Ang II treatment increases the level of MrgD in vivo and vitro, while MrgD ligand Ala alleviates Ang II-induced cardiac hypertrophy [14]. The biological interactions of these two ligands with the MrgD receptor showed that MrgD-mediated signaling is linked to cardiovascular pathophysiology [12, 13]. However, the specific role of MrgD in the cardiovascular system is yet to be elucidated.

Therefore, the present study aimed to investigate the roles of MrgD in regulating cardiac hypertrophy and fibrosis.

## Materials and methods

### Ethics approval and animal care

Six-week-old male C57BL6/J mice and male Sprague–Dawley (SD) rats weighing 200–250 g (Vital River Biological Co., Ltd, Beijing, China) were used for analysis. All procedures were approved by the Experimental Animal Care and Use Committee of Nanjing Medical University and conducted in accordance with the Guide for the Care and Use of Laboratory Animals (NIH publication No. 85–23, revised 1996).

The MrgD-knockout (KO) mouse was constructed by Cyagen (Suzhou, China). Briefly, MrgD flox mice were produced by placing a LoxP sequence at each end of the MrgD DNA sequence to be knocked out. Then, the CAG-CreERT mice were crossed with MrgD floxed mice obtaining Cre^+/–^MrgD^flox/flox^ mice. Genotypes were determined by PCR on genomic DNA from tails using primers distinguishing between the wild‐type and floxed alleles. After that, the six-week-old Cre^+/–^MrgD^flox/flox^ mice received intraperitoneal injection of tamoxifen (20 mg/kg) for five consecutive days. All mice used in the study were on a C57Bl/6 J background.

### AngII infusion

After randomly assignment into groups according to body weight, the animals were subjected to a 4-week infusion of Ang II (Sigma) or saline (solvent control) administered by mini-osmotic pumps (model 2006; ALZET Osmotic Pumps, CA, USA) at an infusion rate of 1.44 mg/kg/day.

### Culture and treatment of cardiomyocytes

Primary neonatal rat cardiomyocytes (NRCM) were isolated from 1–2-day-old newborn SD rats as previously report [15]. Briefly, hearts were excised into 1 mm^3^ pieces and digested in phosphate buffer saline (PBS, Nanjing Bio-Channel Biotechnology Co., Ltd, Nanjing, China) containing Collagenase type II (Worthington Biochemical Co., NJ, USA) and pancreatin (Sigma, MO, USA). Then, around 4 × 10^5^ cells were collected and cultured in 10 cm cell culture dishes with complete Dulbecco’s modified Eagle’s medium (DMEM, Nanjing Bio-Channel Biotechnology Co. Ltd) supplemented with 10% fetal bovine serum (FBS, Nanjing Bio-Channel Biotechnology Co., Ltd) for 1–2 h to allow for the preferential attachment of primary neonatal rat fibroblasts (NRCF). Next, the medium containing cardiomyocytes were removed and re-cultured in another pre-coated culture dishes at 37 °C with 5% CO_2_.

After 2-day culture, NRCM were incubated in serum-free DMEM medium for overnight starvation before the further experiment. Then, NRCF and NRCM were incubated with Ang II (Sigma; 10^–6^ M), phenylephrine (PE; Sigma; 10^–4^ M), isoproterenol (ISO; Sigma; 10^–5^ M) or transforming growth factor-beta (TGF-β; Sigma; 10 ng/mL) for 24 h to induce the pathological phenotype, separately.

For knockdown or overexpression experiments, when the cells achieved 70–80% confluency, they were transfected with serum-free medium containing 1e + 7Tu/ml recombinant adenovirus-MrgD (Ad-MrgD) or adenovirus-shRNA-MrgD (MrgD shRNA) (GeneChem Co., Shanghai, China) for 6-8 h.

We also purchased AC16, the human cardiomyocyte line, from the cell bank of the Institute of Biochemistry and Cell Biology of the Chinese Academy of Sciences (Shanghai, China).

### Intra-myocardial injection

A total of 14 adult SD rats, weighing 200–250 g, were randomly divided into Ad-GFP and Ad-MrgD groups. Each isoflurane (2.5–3.5%)-anesthetized, open-chest rat was subjected to endotracheal intubation without tracheostomy. A microsyringe was used for precise injection. Starting from the apex to the heart, the needle was advanced at an approximately 45° angle into four desired injection locations in the left ventricular myocardium directly and circumferentially. A volume of 5 µl was delivered slowly (over 30 s) in each injection to avoid the leak, and the needle was withdrawn by counter-clockwise rotation promptly. Then, the sternum was closed, and the rats were weaned from the ventilator. After another 4-week breeding, these animals were euthanized, and the left ventricles excised for follow-up studies.

### Echocardiography

Mice transthoracic echocardiography was performed under isoflurane anesthesia (1.5–2.5%) using an ultrasound system (Vevo 2100, VisualSonics, Toronto, Canada) with a 21-MHz probe. The measurements over three consecutive cardiac cycles were averaged.

### Real-time polymerase chain reaction (RT-PCR)

Total RNA was isolated from cells or tissues using TRIzol (Invitrogen Inc., San Diego, CA, USA), and 0.5 μg was reverse transcribed into cDNA using PrimeScript™ RT reagents kit (TaKaRa, Kyoto, Japan). Real-time PCR reactions were performed on an ABI Prism 7900 system. The primers are listed in Table S1. The relative mRNA level was expressed as 2^−ΔΔCt^.

### Western blotting

Around 4 × 10^5^ cultured cells or 30 μg heart tissues were sonicated in RIPA lysis buffer and homogenized. The supernatant obtained by centrifugation at 12000 g for 10 min at 4°C. The nuclear protein was extracted using NucBuster™ protein extraction kit (Novagen®, Rockland, DE, USA), according to manufacturer’s protocol. Approximately 30 μg protein was separated in 10% SDS-PAGE gel (Beyotime Biotechnology, Nanjing, China) and transferred to polyvinylidene fluoride (PVDF) membrane (Merck-Millipore, Shanghai, China). After blocking with 5% bovine serum albumin for 2 h at room temperature, the membranes were incubated overnight at 4°C with primary antibodies against MrgD (1:1000 dilution; Abcam), alpha-smooth muscle actin (α-SMA; 1:1000; Cell Signaling Technology, Danvers, MA, USA), transforming growth factor-beta (TGF-β; 1:1000 dilution; Beyotime Biotechnology, China), and Collagen I (1:1000 dilution; Abcam, MA, USA); Tubulin (1:1000 dilution; Abcam); GAPDH (1:1000 dilution; Abcam) or Lamin B (1:1000 dilution; Cell Signaling Technology) was used as an internal control.

### Immunofluorescence

Cells were fixed with 4% paraformaldehyde (PFA) for 15 min at room temperature and incubated with a blocking solution containing 10% bovine serum albumin (BSA) for 1 h. Then, the cells were incubated with primary antibody against MrgD (1:200; Alomone labs, Israel) and actin (Abcam) at 4°C overnight, followed by the corresponding secondary antibodies (Jackson ImmunoResearch, West Grove, PA, USA) for 2 h at room temperature. Then, 4’,6-diamidino-2-phenylindole (DAPI; Life Technologies Co., Grand Island, NY, USA) was used to counterstain the nucleus. The fluorescent cell images were captured using a fluorescence microscope (Carl Zeiss GmbH, Oberkochen, Germany). The experiments were performed in triplicate.

### Hematoxylin–eosin (HE) and Wheat germ agglutinin (WGA) staining

Heart Sects. (5 µm) were examined by HE staining (Service Biological Technology Co., Ltd, Wuhan, China) according to the provided procedures. The images were observed under a light microscope (Olympus Corporation, Tokyo, Japan), and were analyzed using Image-Pro Plus software (version 6.0; Media Cybernetics, Inc., Bethesda, MD, USA).

Heart Sects. (5 µm) were stained using WGA (Invitrogen Inc., CA, USA) to measure the cross-sectional area of cardiomyocytes. The images were observed under a confocal microscope (Carl Zeiss GmbH, Oberkochen, Germany), and were analyzed with Image-Pro Plus software. Briefly, cardiomyocyte size was evaluated by measuring the cross-sectional area along the short axis of cardiomyocytes.

### Masson’s and sirius red staining

Heart Sects. (5 µm) were stained with Masson’s and Picrosirius red staining (Service Biological Technology Co., Ltd, Wuhan, China) according to the provided procedures. Then, the sections were observed under a Zeiss fluorescence upright microscope (Carl Zeiss GmbH, Oberkochen, Germany). More than 3 images were taken randomly. Next, the Image-Pro Plus software was used to quantify the percentage of myocardial fibrosis. 

### Coimmunoprecipitation (Co-IP)

We first extracted the nuclear protein of cells receiving different treatments. Next, the Protein A/G Agarose (P2055; Beyotime) was incubated with 1 μg antibody against rabbit-IgG (Sc-2027, Santa Cruz Biotechnology) or MrgD for 2 h at room temperature with shaking to form the immune complex. Then, the protein samples were incubated with the immune complex overnight at 4°C under constant rotation. After rinsed with TBS three times, the beads were resuspended with SDS-PAGE sample loading buffer (P0015L; Beyotime), which were then subjected to SDS-PAGE. The levels of phosphorylation-P38 were detected in the immunoprecipitated samples using antibody against p-P38 (Cat. 4511, Cell Signaling Technology).

### Measurement of cAMP in cell lysates

The Direct cAMP ELISA kit (USCN, Wuhan, China) was used to detect the cAMP concentration in cell lysates. Briefly, 100 µl of cell lysate or cAMP standard solution was added in the Goat Anti-Rabbit IgG antibody-coated plate wells (1 plate, 96 wells). Then, the binding solution containing 50 µl cAMP-Alkaline Phosphatase (AP) Conjugate and 50 µl EIA Rabbit Anti-cAMP antibody was added in each well. Following a 2-h incubation at 400 rpm at room temperature, the plate was aspirated. Next, each well was rinsed with Wash Buffer for 3 times. After that, 200 µl p-Nitrophenyl Phosphate Substrate Solution was added in the well. After a 1-h incubation at room temperature, 50 µl Stop Solution was added into the well to stop the reaction. Immediately, the cAMP concentration was determined by measuring the absorbance at 405 nm.

### Statistical analyses

Data are presented as mean ± standard error of the mean (SEM) using GraphPad Prism 7.0 (GraphPad Software Inc., San Diego, CA, USA). The statistical significance among multiple groups was evaluated by one-way analysis of variance (ANOVA) with the Bonferroni post-hoc test. And t-test was performed to compare the differences between the 2 groups. A two-tailed *P*-value < 0.05 was considered statistically significant.

## Results

### Expression of MrgD in different pathological models

**Fig. 1:**
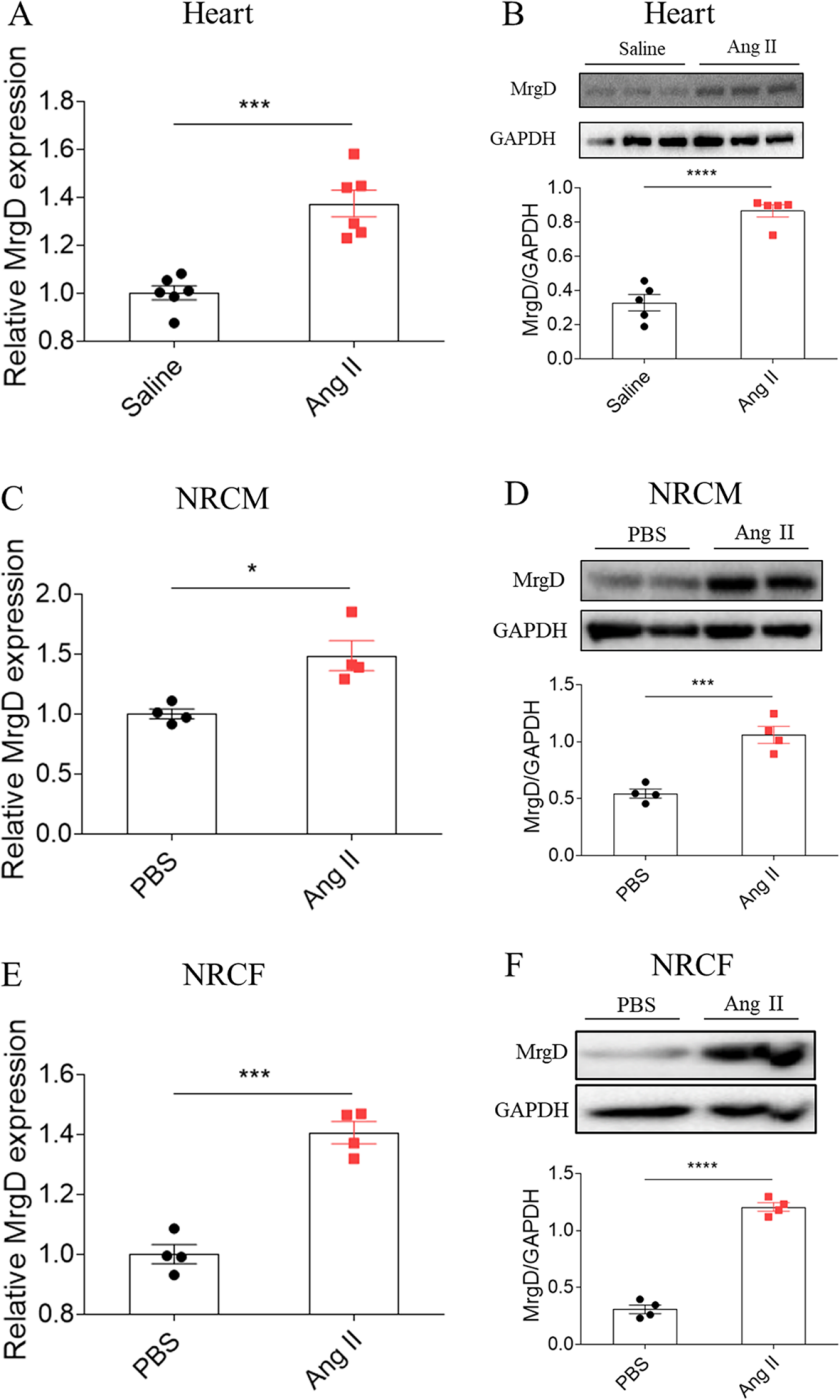
Expression of MrgD in different pathological models. **A**-**B**, mRNA and protein expressions of MrgD were increased in the heart of Ang II-treated mice. **C**-**D**, mRNA and protein expressions of MrgD were increased in the Ang II-treated NRCM. **E**–**F**, mRNA and protein expressions of MrgD were increased in the Ang II-treated NRCF. The data are expressed as mean ± SEM. *n* = 4, 5 or 6 biological replicates per group. ^ns^*P* > 0.05, ^*^P < 0.05, ^**^P < 0.01, ^***^P < 0.001, ^****^P < 0.0001

First, we found that MrgD expression was higher in heart tissues of Ang II-infused mice than those of the control group (Fig. 1A-B). As shown in Fig. 1C-D, after the stimulation of Ang II, MrgD expression augmented in NRCM. Its expression was also increased following the induction of Ang II than that in the control group in NRCF (Fig. 1E-F). Besides, MrgD expression was also detected in other cardiac remodeling models, such as TAC mice (Figure S1A-S1B). Morever, the expression of MrgD was elevated in the PE or ISO-treated NRCM (Figure S1C-S1F), and TGF-β-treated NRCF (Figure S1G-S1H).

### Effects of MrgD on hypertrophy of NRCM and fibrosis of NRCF

**Fig. 2:**
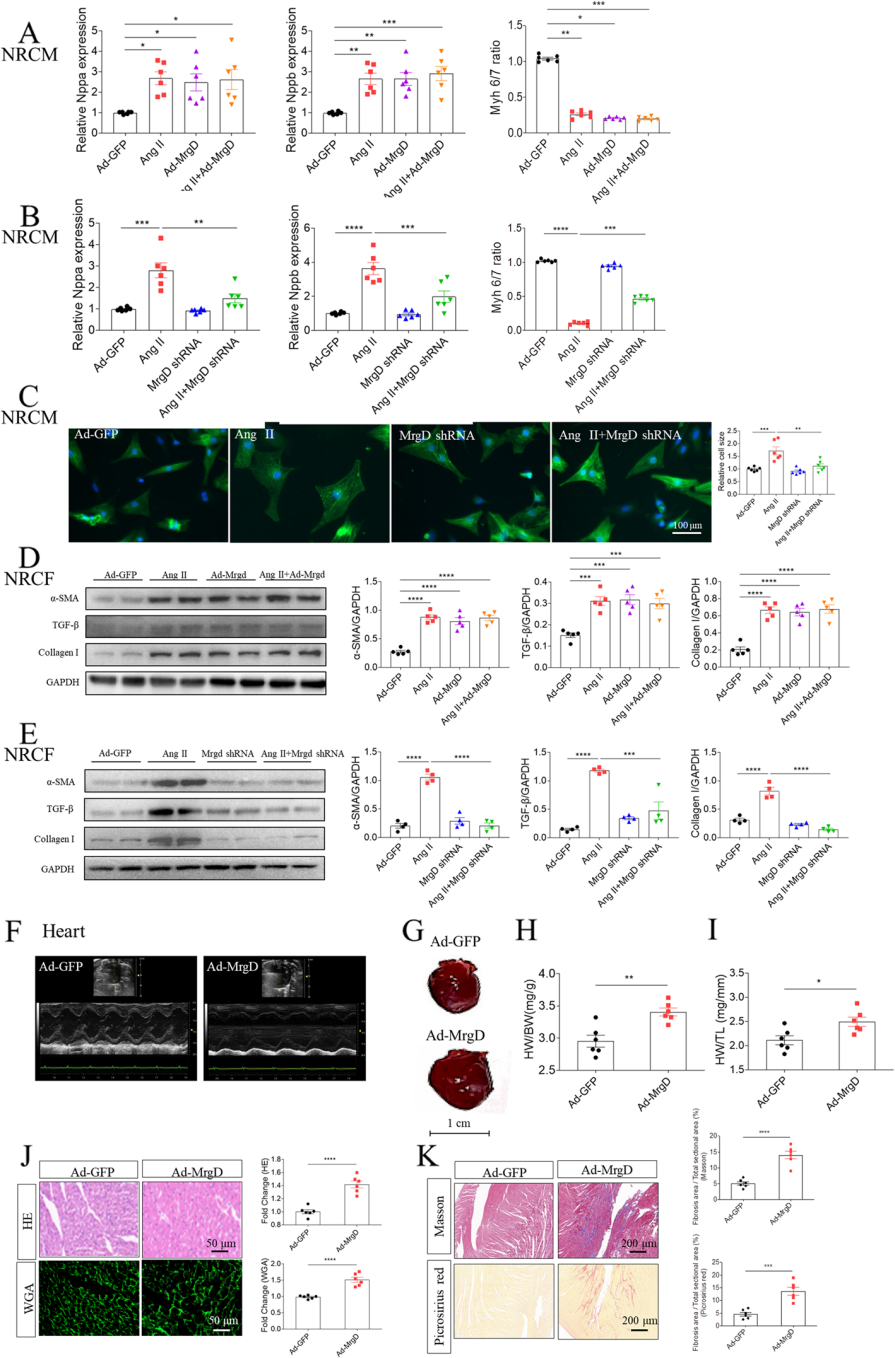
Effects of MrgD upregulation or downregulation on cardiac hypertrophy and fibrosis. **A**, MrgD overexpression altered the mRNA levels of Nppa, and Nppb, as well as the ratio of Myh 6/7 in NRCM. **B**, MrgD downregulation reversed the mRNA changes of Nppa, and Nppb in NRCM induced by Ang II. **C**, MrgD knockdown attenuated the increase in the size of NRCM induced by AngII. **D**, MrgD overexpression increased the protein levels of α-SMA, TGF-β and collagen I in NRCF. **E**, MrgD downregulation reversed the increase in α-SMA, TGF-β and collagen I induced by AngII in NRCF. **F**, Echocardiographic images of hearts. **G**-**I**, Cardiac size, HW/BW and HW/TL were increased after MrgD overexpression in the heart. **J**, MrgD overexpression increased the cross-sectional area of cardiomyocytes in the rats as indicated by HE and WGA staining. **K**, MrgD overexpression increased the cardiac fibrosis level in the rats, as indicated by masson and picrosirius red staining. The results are expressed as mean ± SEM. *n* = 4, 5 or 6 biological replicates per group. ^ns^*P* > 0.05, ^*^P < 0.05, ^**^P < 0.01, ^***^P < 0.001, ^****^P < 0.0001

In order to investigate the functional role of MrgD, we constructed Ad-MrgD and MrgD shRNA. First, we verified the transfection efficiency of Ad-MrgD, and MrgD shRNA in vitro, including NRCM (Figure S2A-S2B) and NRCF (Figure S2C-S2D). After transfection, we found that MrgD overexpression increased mRNA levels of Nppa, and Nppb, and reduced the Myh 6/7 ratio in NRCM (Fig. 2A). Interestingly, MrgD overexpression did not further enhance Ang II-induced changes of Nppa, Nppb, and Myh 6/7 ratio (Fig. 2A), which may be accounted for dose saturation effect. Then, the increase of Nppa, and Nppb mRNA levels, and the decrease of Myh 6/7 ratio were alleviated by silencing MrgD (Fig. 2B). Next, MrgD knockdown attenuated Ang II-induced increase in the size of NRCM (Fig. 2C). Meanwhile, MrgD overexpression increased the protein levels of α-SMA, TGF-β and collagen I (Fig. 2D), and the mRNA levels of Acta2, TGF-β, COL1A1 (Figure S3A) in NRCF. Also, MrgD knockdown ameliorated Ang II-induced increases in the protein of α-SMA, TGF-β and collagen I (Fig. 2E), and mRNA of Acta2, TGF-β and COL1A1 (Figure S3B) in NRCF. The above results showed that MrgD may play a regulatory role in cardiac hypertrophy and fibrosis.

### Effects of MrgD on cardiac hypertrophy and fibrosis of mice in vivo

In order to further establish the effects of MrgD on cardiac remodeling in vivo, we first established a cardiac MrgD overexpression model using intra-myocardial injection. MrgD expression was elevated in the heart after Ad-MrgD microinjection successfully (Figure S4A-S4C). Then, the results of echocardiography after 4-week breeding showed that the LVEF and LVFS were significantly reduced in the rats after MrgD overexpression, indicating that MrgD overexpression may deteriorate cardiac function of rats (Fig. 2F and Table 1). The cardiac size (Fig. 2G), heart weight/body weight (HW/BW; Fig. 2H), and HW/tibial length (HW/TL; Fig. 2I) were also increased as a response to MrgD overexpression in the heart. Histopathologically, the area of the cardiomyocytes was higher in the heart of MrgD overexpression group than that in the GFP group via HE or WGA staining (Fig. 2J). The mRNA levels of Nppa, and Nppb were increased, while Myh 6/7 ratio was reduced in the heart of rats with MrgD overexpression (Figure S4D). Moreover, the cardiac fibrosis was increased after MrgD overexpression via masson or picrosirius red staining (Fig. 2K). MrgD overexpression in the heart increased the mRNA and protein levels of TGF-β and COL1A1 (Figure S4E-S4F) in the heart of rats as well. The above results indicated the pro-remodeling effects of MrgD overexpression in the hearts.

**Table 1:** Cardiac function was reduced after MrgD overexpression in the hearts of rat

Variables	Ad-GFP (*n* = 6)	Ad-MrgD (*n* = 6)
LVEF (%)	72.15 ± 0.6543	54.39 ± 1.084^***^
LVFS (%)	42.57 ± 0.1386	29.33 ± 0.822^***^
LV mass (mg)	1048.07 ± 15.20	1131.80 ± 20.61^*^
LVIDs (mm)	4.177 ± 0.1515	5.735 ± 0.2772^ ns^
LVIDd (mm)	7.274 ± 0.1703	8.116 ± 0.2412^*^
LVAW;s (mm)	3.208 ± 0.1241	2.780 ± 0.1428^*^
LVAW;d (mm)	1.809 ± 0.07785	1.819 ± 0.07617^ ns^
LVPW;s (mm)	3.137 ± 0.09524	2.743 ± 0.07900^**^
LVPW;d (mm)	2.094 ± 0.1134	1.784 ± 0.06935^*^
Heart weight (mg)	1063 ± 25.21	1523 ± 63.62^****^
Body weight (g)	328.3 ± 17.31	302.6 ± 13.49^ ns^
Tibia length (mm)	48.07 ± 0.3029	48.08 ± 0.2762^ ns^

Echocardiographic data of mice receiving intra-myocardial injection of Ad-MrgD or GFP. The results are expressed as the mean ± SEM (*LVEF* Left ventricular ejection fraction, *LVFS* Left ventricular fractional shortening, *LVIDs* LV internal diameters at end systole, *LVIDd* LV internal diameters at end diastole, *LVAW;s* Left ventricular systolic anterior wall, *LVAW;d* Left ventricular diastolic anterior wall, *LVPW;s* Left ventricular systolic posterior wall, *LVPW;d* Left ventricular diastolic posterior wall)

^ns^*P > *0.05, ^*^P < 0.05, ^**^P < 0.01, ^***^P < 0.001, ^****^P < 0.0001

**Fig. 3:**
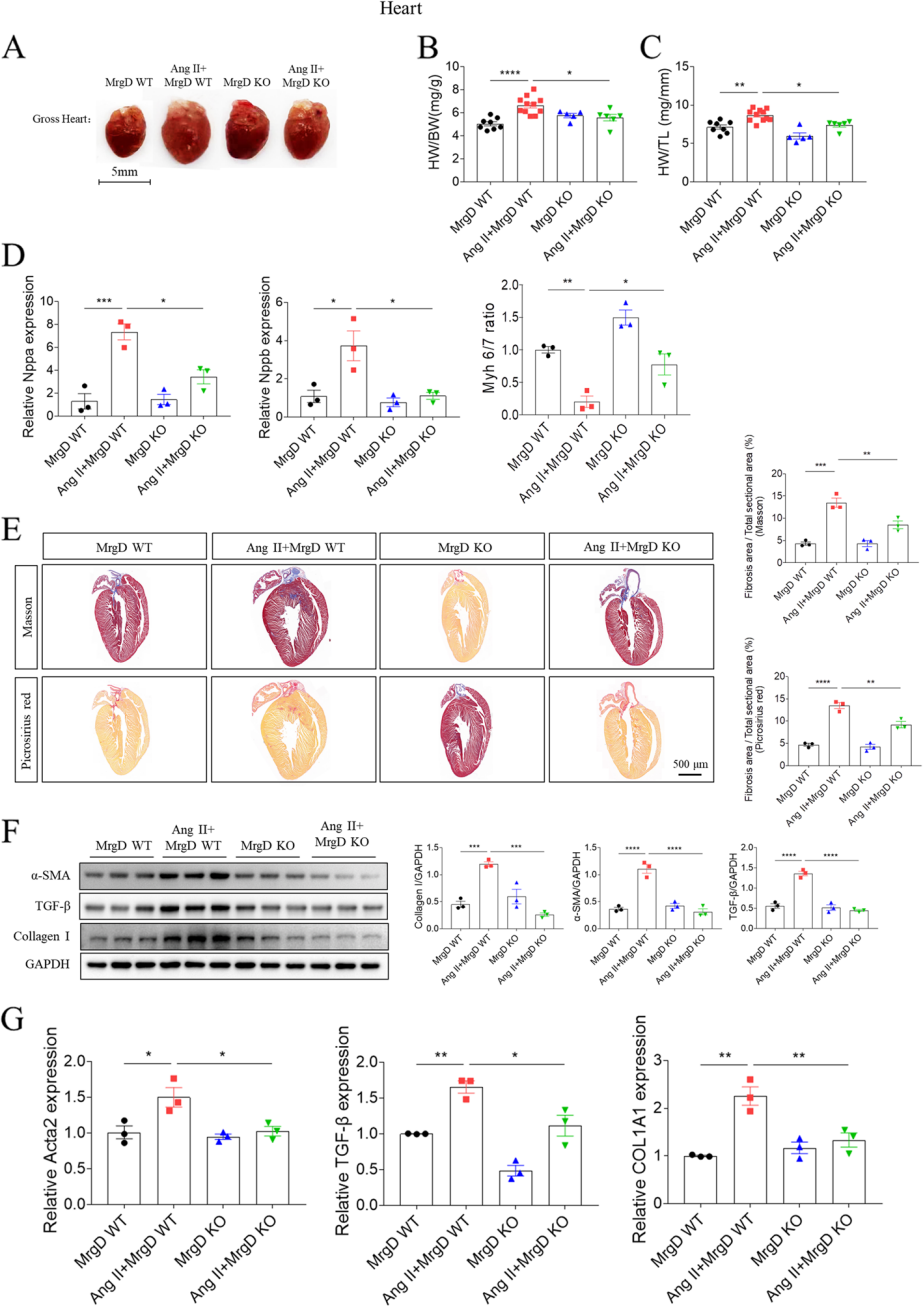
Effects of MrgD KO on cardiac hypertrophy and fibrosis. **A**-**C**, Cardiac size, HW/BW and HW/TL were decreased after MrgD KO in the heart compared with those in the Ang II + MrgD WT group. **D**, the mRNA levels of Nppa, and Nppb, as well as the ratio of Myh 6/7 in the hearts from different groups. **E**, MrgD KO decreased the cardiac fibrosis level in the Ang II-induced rats, as indicated by masson and picrosirius red staining. **F**-**G**, the protein and mRNA levels of α-SMA, TGF-β and collagen I in the hearts from different groups. The results are expressed as mean ± SEM. *n* = 6 biological replicates per group. ^ns^*P* > 0.05, ^*^P < 0.05, ^**^P < 0.01, ^***^P < 0.001, ^****^P < 0.0001

Next, we established a MrgD-KO mouse model to examine the role of MrgD in Ang II-induced cardiac hypertrophy and fibrosis. The MrgD expression was reduced in the hearts of KO mice (Figure S5A-S5C). After 4-week-induction of Ang II, the mice in the Ang II + MrgD WT group had a worse cardiac function with the enlarged gross hearts, increased ratio of HW/TL and HW/BW, which were improved in MrgD KO mice (Table 2 and Fig. 3A-C). The similar trend was also seen in the mRNA expression of Nppa, and Nppb, as well as the ratio of Myh 6/7, indicating the protective role of MrgD knockdown in Ang II-induced cardiac hypertrophy (Fig. 3D). Further, the results of the quantitative analysis of masson and picrosirius red staining in the heart sections showed that remarked collagen deposition in LV tissues was visualized in the Ang II + MrgD WT group, while mice in the Ang II + MrgD KO group displayed less collagen accumulation than those in the Ang II + MrgD WT group (Fig. 3E). Also, we found that MrgD knockdown could ameliorate Ang II-induced increased α-SMA, TGF-β and collagen I protein (Fig. 3F), and Acta2, TGF-β and COL1A1 mRNA (Fig. 3G) expression. The above results indicated that MrgD knockdown may exert a protective effect against Ang II-induced myocardial hypertrophy and fibrosis.

**Table 2:** MrgD KO improved AngII-induced cardiac dysfunction in the mice

Variables	MrgD WT (*n* = 8)	AngII + MrgD WT (*n* = 8)	MrgD KO (*n* = 8)	AngII + MrgD KO (*n* = 8)
LVEF (%)	75.40 ± 2.278	75.34 ± 1.594^ ns^	78.31 ± 2.395	68.13 ± 5.952^ ns^
LVFS (%)	43. 76 ± 2.108	43.17 ± 1.641^ ns^	46.10 ± 2.309	38.12 ± 4.133^ ns^
LV mass (mg)	135.1 ± 6.036	161.9 ± 5.127 ^*^	108.3 ± 4.645	139.8 ± 9.153^ ns^
LVIDs (mm)	2.004 ± 0.1009	1.793 ± 0.06207^ ns^	1.786 ± 0.1073	2.116 ± 0.2544^ ns^
LVIDd (mm)	3.551 ± 0.05798	3.162 ± 0.08756 ^*^	3.304 ± 0.07619	3.319 ± 0.1373^ ns^
Volume;s (ml)	13.25 ± 1.591	9.831 ± 0.8532^ ns^	9.798 ± 1.460	16.64 ± 5.265^ ns^
Volume;d (ml)	52.88 ± 1.997	40.26 ± 2.743 ^*^	44.46 ± 2.464	45.38 ± 4.632^ ns^
LVAW;s (mm)	1.665 ± 0.05349	1.845 ± 0.05388^ ns^	1.533 ± 0.02593	1.630 ± 0.05772 ^#^
LVAW;d (mm)	1.102 ± 0.03870	1.285 ± 0.05180 ^*^	0.9819 ± 0.03872	1.114 ± 0.02342 ns
LVPW;s (mm)	1.428 ± 0.02349	1.708 ± 0.05019 ^***^	1.342 ± 0.05937	1.459 ± 0.04320 ^#^
LVPW;d (mm)	0.9150 ± 0.02328	1.253 ± 0.06023 ^****^	0.8911 ± 0.01179	1.060 ± 0.05403 ^#^
Heart weight (mg)	100.4 ± 1.547	141.2 ± 5.014 ^****^	100.3 ± 3.376	118.1 ± 4.179 ^##^
Body weight (g)	20.04 ± 0.6955	19.65 ± 0.9044^ ns^	17.94 ± 0.7117	19.55 ± 0.9614^ ns^
Tibia length (mm)	16.00 ± 1.2080	17.81 ± 0.8991^ ns^	17.07 ± 1.283	16.02 ± 0.5746^ ns^

Echocardiographic data of mice from different groups. The results are expressed as the mean ± SEM (*LVEF* Left ventricular ejection fraction, *LVFS* Left ventricular fractional shortening, *LVIDs* LV internal diameters at end systole, *LVIDd* LV internal diameters at end diastole, *Volume;s* Volume at end systole, *Volume;d* Volume at end diastole, *LVAW;s* Left ventricular systolic anterior wall, *LVAW;d* Left ventricular diastolic anterior wall, *LVPW;s* Left ventricular systolic posterior wall, *LVPW;d* Left ventricular diastolic posterior wall). (^*^: vers MrgD WT group; ^#^: vers AngII + MrgD WT group)

^ns^*P > *0.05, ^*^P < 0.05, ^***^P < 0.001, ^****^P < 0.0001; ^#^*P *< 0.05, ^##^*P *< 0.01

### Effects of Ala on NRCM hypertrophy and NRCF fibrosis

We next investigate the role of Ala, the endogenous ligand of MrgD, in Ang II-induced cardiac remodeling to further clarity the cardiac regulatory role of MrgD. Here, we treated NRCM or NRCF with Ala after Ang II stimulation. First, we found that Ala treatment reversed Ang II-induced changes of Nppa, and Nppb mRNA expression, as well as Myh 6/7 ratio in NRCM (Figure S6A). Ala treatment also inhibited Ang II-induced the increases of Acta2, TGF-β and COL1A1 mRNA (Figure S6B), and α-SMA, TGF-β and collagen I protein expression in NRCF (Figure S6C). Morever, we found Ala attenuated Ad-MrgD-induced cardiac hypertrophy (Figure S7A) and fibrosis (Figure S7B) in vitro, indicating that Ala may be an antagonist that combines with its receptor MrgD for the downstream actions.

**Fig. 4:**
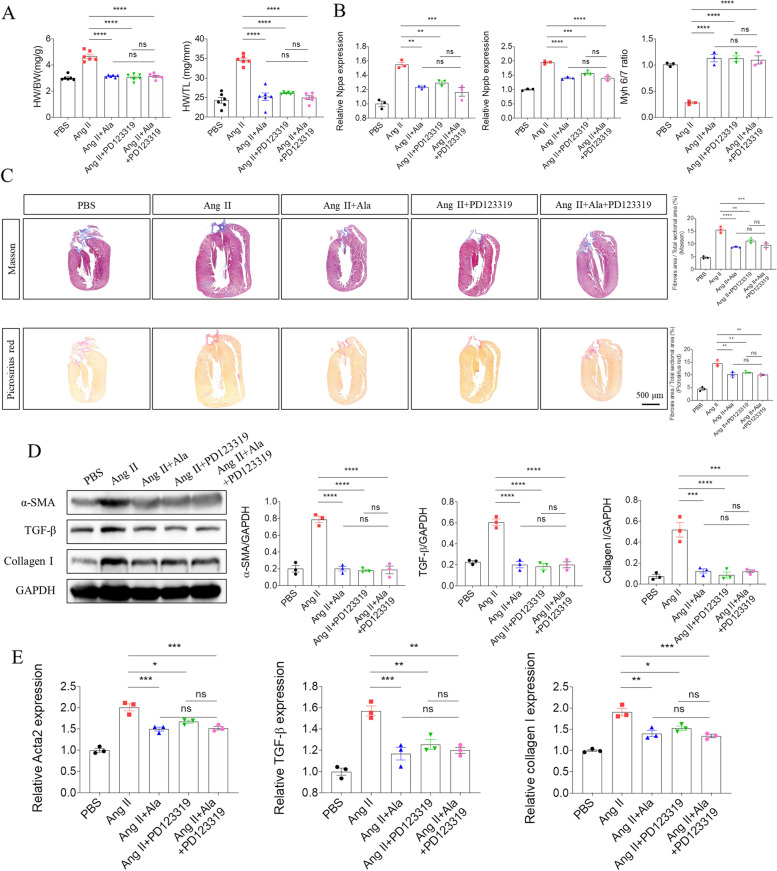
Effects of Ala or PD123319 on Ang II-induced cardiac remodeling. **A**, HW/BW and HW/TL were decreased after Ala or PD12339 pretreatment in the heart compared with those in the Ang II group. **B**, the mRNA levels of Nppa and Nppb, as well as the ratio of Myh 6/7 in the hearts from different groups. **C**, Ala or PD12339 pretreatment decreased the cardiac fibrosis level in the Ang II-induced rats, as indicated by masson and picrosirius red staining. **D**-**E**, the protein and mRNA levels of α-SMA, TGF-β and collagen I in the hearts from different groups. The results are expressed as mean ± SEM. *n* = 3 biological replicates per group. ^ns^*P* > 0.05, ^*^P < 0.05, ^**^P < 0.01, ^***^P < 0.001, ^****^P < 0.0001

In order to better verify our above conclusion, we next investigated whether either Ala or PD123319 (MrgD receptor blocker) would block the detrimental effects of MrgD overexpression. In vivo, after intra-myocardial injection of Ad-MrgD, the rats received Ala (50 μg/kg/day[14]) or PD123319 (3 mg/kg/day) or the mixed solution combined with Ala (50 μg/kg/day) and PD123319 (3 mg/kg/day) intraperitoneally daily for the consecutive 4 weeks. After harvest, we found that both Ala and PD123319 improved Ad-MrgD-induced cardiac function in rats (Table 3 and Fig. 4A). The similar trend was also seen in the mRNA expression of Nppa, and Nppb, as well as the ratio of Myh 6/7 (Fig. 4B). Besides, the histopathological results showed the inhibitory effects of Ala or PD123319 on Ad-MrgD-induced collagen deposition in heart tissues (Fig. 4C). Also, either Ala or PD123319 prevented Ad-MrgD-induced increased α-SMA, TGF-β and collagen I protein (Fig. 4D), and Acta2, TGF-β and COL1A1 mRNA (Fig. 4E) expression. However, no obvious difference in myocardial hypertrophy and fibrosis was found among Ad-MrgD + Ala, Ad-MrgD + PD123319, and Ad-MrgD + Ala + PD123319 group. Morever, in NRCM and NRCF, we found that either Ala or PD123319 ameliorated pro-hypertrophic and pro-fibrotic effects of MrgD overexpression (Figure S8A-S8B). Also, no additional effects were found when combind Ala and PD123319 in vitro.

**Table 3:** PTX pre-treatment blocked the cardioprotective effects of silencing MrgD on Ang II-induced cardiac dysfunction in the rat

Variables	PBS (*n* = 6)	Ang II (*n* = 6)	Ang II + MrgD shRNA (*n* = 6)	Ang II + MrgD shRNA + PTX (*n* = 6)
LVEF (%)	76.87 ± 1.034	78.14 ± 1.546^ ns^	79.88 ± 1.113	75.85 ± 1.678^ ns^
LVFS (%)	47.30 ± 0.9982	48.34 ± 1.508^ ns^	50.15 ± 1.070	46.09 ± 1.567^ ns^
LV mass (mg)	1137 ± 42.25	1630 ± 108.1 ^***^	1279 ± 58.35	1526 ± 56.63^ ns^
LVIDs (mm)	4.291 ± 0.1270	3.793 ± 0.1080 ^*^	3.891 ± 0.1251	3.828 ± 0.0694^ ns^
LVIDd (mm)	8.144 ± 0.2041	7.346 ± 0.0865 ^**^	7.753 ± 0.0786	7.333 ± 0.1876^ ns^
LVAW;s (mm)	2.858 ± 0.05936	4.101 ± 0.2250 ^****^	3.131 ± 0.06436	3.670 ± 0.1059 ^#^
LVAW;d (mm)	1.786 ± 0.04700	2.573 ± 0.1292 ^****^	2.123 ± 0.06021	2.427 ± 0.06379 ^#^
LVPW;s (mm)	2.813 ± 0.07173	3.749 ± 0.1851 ^****^	2.822 ± 0.1032	3.440 ± 0.07458 ^##^
LVPW;d (mm)	1.812 ± 0.05791	2.567 ± 0.1251 ^****^	2.143 ± 0.08041	2.497 ± 0.04042 ^#^
Heart weight (mg)	1196 ± 41.28	1576 ± 47.00 ^***^	1315 ± 76.10	1575 ± 36.22 ^#^
Body weight (g)	360.0 ± 5.971	349.3 ± 14.03^ ns^	346.7 ± 8.065	360.0 ± 10.41^ ns^
Tibia length (mm)	48.06 ± 0.2562	48.07 ± 0.2337^ ns^	47.87 ± 0.2186	47.78 ± 0.1833^ ns^

Echocardiographic data of mice from different groups. The results are expressed as the mean ± SEM (*LVEF* Left ventricular ejection fraction, *LVFS* Left ventricular fractional shortening, *LVIDs* LV internal diameters at end systole, *LVIDd* LV internal diameters at end diastole, *Volume;s* Volume at end systole, *Volume;d* Volume at end diastole, *LVAW;s* Left ventricular systolic anterior wall, *LVAW;d* Left ventricular diastolic anterior wall, *LVPW;s* Left ventricular systolic posterior wall, *LVPW;d* Left ventricular diastolic posterior wall). (^*^: vers PBS group; ^#^: vers Ang II + MrgD shRNA group)

^ns^*P *> 0.05, ^*^P < 0.05, ^**^P < 0.01, ^***^P < 0.001, ^****^P < 0.0001; ^#^*P *< 0.05, ^##^*P *< 0.01

### Nuclear import of MrgD in NRCM and NRCF

**Fig. 5:**
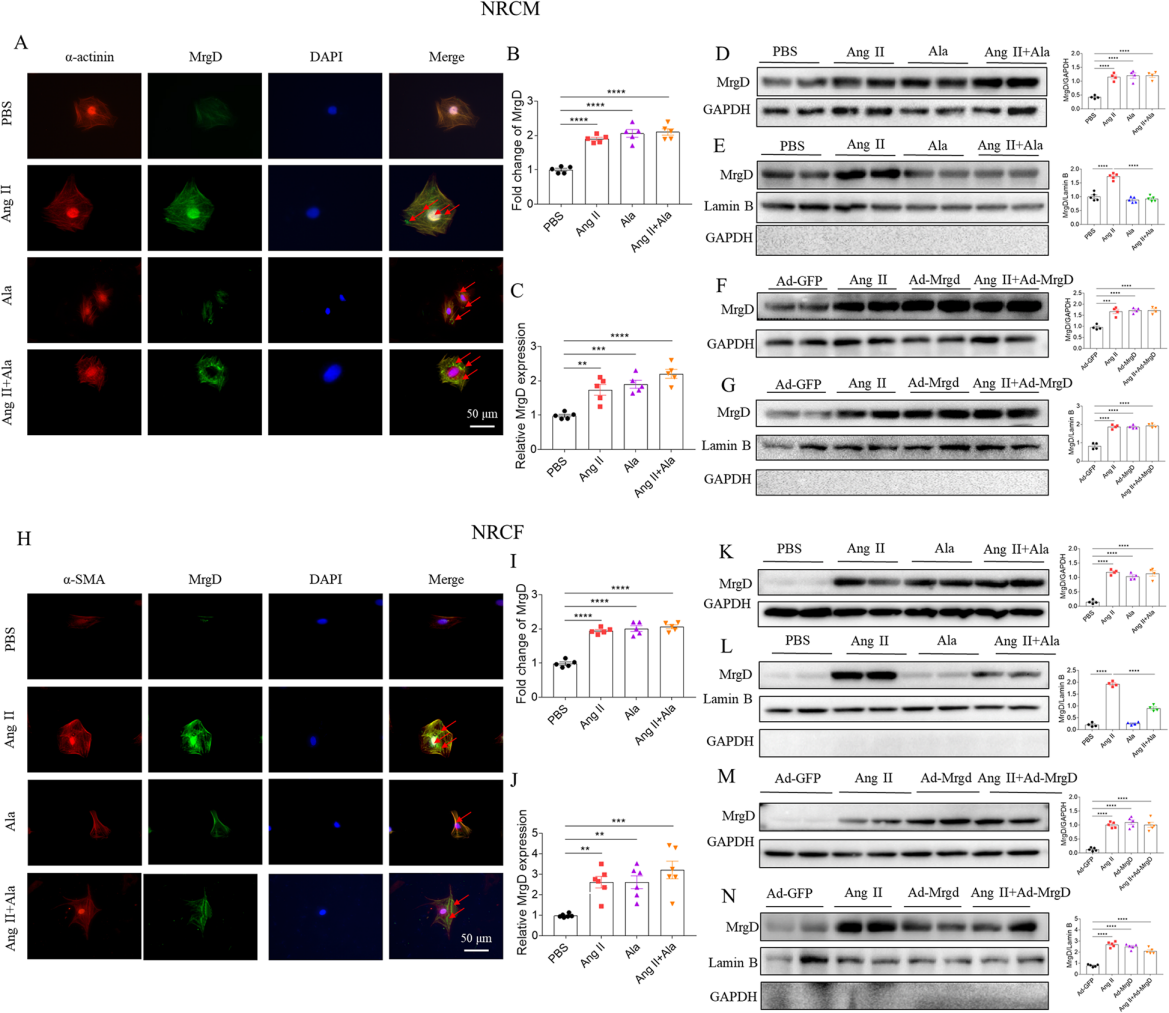
Effects of Ala on MrgD expression in NRCM and NRCF. **A**-**B**, MrgD was detected in the membrane, cytoplasm, and nuclear region of NRCM, and the number of NRCM with positive MrgD was increased in Ala or Ang II groups. **C**, Ala or Ang II increased the mRNA level of MrgD in NRCM. **D**, Ala or Ang II treatment increased the MrgD protein level in NRCM. **E**, Ala inhibited the increase in MrgD level in the nuclei of NRCM. **F**-**G**, MrgD overexpression increased the MrgD levels in the total protein or nuclei of NRCM. **H**-**I**, MrgD was detected in the membrane, cytoplasm, and nuclear region of NRCF, and the number of NRCF with positive MrgD was increased in Ala or Ang II groups. **J**, Ala or Ang II increased the mRNA level of MrgD in NRCF. **K**, Ala or Ang II treatment increased the MrgD level in total protein of NRCF. **L**, Ala inhibited the increase in MrgD level in the nuclei of NRCF. **M**–**N**, MrgD overexpression increased the MrgD protein level or that in nuclei of NRCF. The data are expressed as mean ± SEM. *n* = 4, 5 or 6 biological replicates per group. ^ns^*P* > 0.05, ^*^P < 0.05, ^**^P < 0.01, ^***^P < 0.001, ^****^P < 0.0001

In order to investigate the downstream mechanisms accounting for the different regulatory effects of MrgD and Ala towards cardiac hypertrophy and fibrosis, we detected the expression of MrgD in the membrane, cytoplasm, and nuclear region of NRCM and NRCF. First, the fluorescence intensity of MrgD-postive NRCM was increased in Ala or Ang II groups. (Fig. 5A-B). Moreover, the mRNA (Fig. 5C) and total protein expression (Fig. 5D) of MrgD was increased in NRCM treated with Ala or Ang II. Notably, Ang II elevated the translocation of MrgD into the nuclei of NRCM, while Ala inhibited this increase (Fig. 5E). In addition, MrgD overexpression increased both the MrgD total protein (Fig. 5F) and nuclei protein expression (Fig. 5G).

Meanwhile, the increased fluorescence intensity o (Fig.  5H-I). The mRNA and total protein expression of MrgD was also increased in NRCF as a response to Ala or Ang II treatment (Fig. 5J-K). Then, Ang II but not Ala increased the MrgD protein expression in the nuclei of NRCF, which was abolished by Ala treatment (Fig. 5L). In addition, MrgD overexpression increased both the MrgD total protein (Fig. 5F) and nuclei protein expression in NRCM (Fig. 5G) and NRCF (Fig. 5N).

In AC16 human ventricular myocytes, we obseved the same results. Either Ang II or Ala increased MrgD expression (Figure S9A). Besides, Ala pretreatment reversed Ang II-induced nuclear import of MrgD (Figure S9B). Then, Ala or silencing MrgD did reverse Ang II-induced changes of cardiac fetal genes, while MrgD overexpression alone mimicked the pro-hypertrophic effects of Ang II (Figure S9C-S9D).

### Effects of Ala and MrgD on Gα_i_ in NRCM and NRCF

**Fig. 6:**
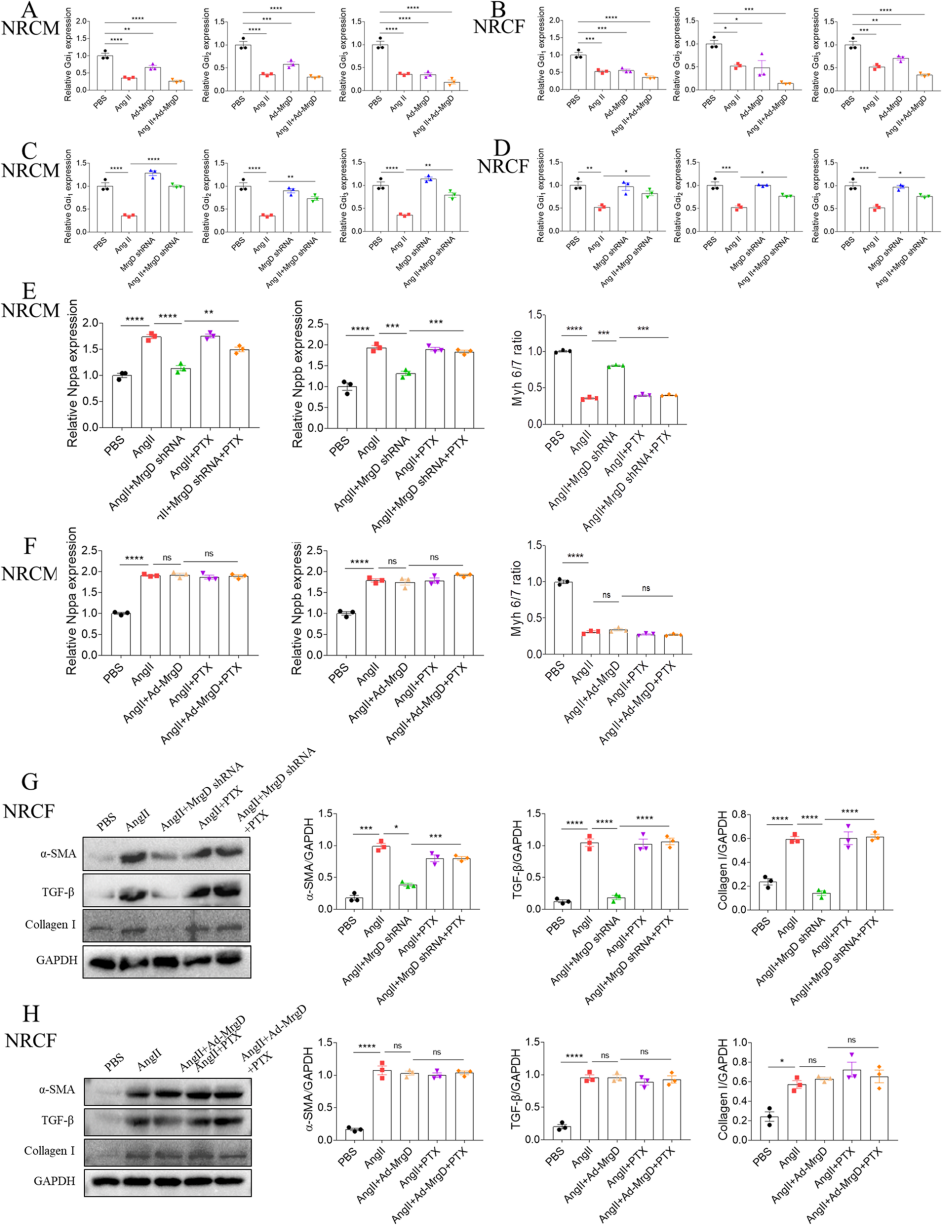
Effects of MrgD on Gα_i_ in NRCM and NRCF. **A**-**B**, MrgD onerexpression inhibited Ang II-induced mRNA expression levels of Gα_i1_, Gα_i2_ and Gα_i3_ in NRCM and NRCF. **C**-**D**, The decreases of Gα_i1_, Gα_i2_ and Gα_i3_ induced by Ang II in NRCM and NRCF were reversed by MrgD knockdown. **E**, PTX pretreatment blocked the protective effects of MrgD shRNA on Ang II-induced fetal genes expression. **F**, Ad-MrgD has no extra effects on enhancing Ang II-induced the changes of fetal genes. **G**, PTX pretreatment blocked the protective effects of MrgD shRNA on Ang II-induced the increases of the α-SMA, TGF-β and collagen I in NRCF. **H**, Ad-MrgD has no extra effects on enhancing Ang II-induced the changes of the α-SMA, TGF-β and collagen I in NRCF. The data are expressed as mean ± SEM. *n* = 3 biological replicates per group. ^ns^*P* > 0.05, ^*^P < 0.05, ^**^P < 0.01, ^***^P < 0.001, ^****^P < 0.0001

Further, we found that NRCMs or NRCFs treated with Ala alone presented higher intracellular cAMP levels than those in the control group (Figure S10A-S10B). Meanwhile, Ala blocked Ad-MrgD-induced decline in intracellular cAMP levels in NRCMs or NRCFs (Figure S10A-S10B). Since the fact that the transcriptional activity and protein expression of Gαi can be regulated by intracellular cAMP concentrations and by agonists such as Ang II and tumour necrosis factor α in vitro [16, 17], we next detected the expression of Gα_i_ in NRCM from different groups. The results showed that Ala treatment reversed Ang II-induced downregulation of Gα_i1_, Gα_i2_ and Gα_i3_ mRNA expression in NRCM and NRCF (Figure S11A-S11AB). Besides, MrgD overexpression reduced Gα_i1_, Gα_i2_ and Gα_i3_ mRNA expression in NRCM (Fig. 6A) and NRCF (Fig. 6B), while MrgD knockdown inhibited Ang II-induced decreases of Gα_i1_, Gα_i2_ and Gα_i3_ mRNA expression in NRCM (Fig. 6C) and NRCF (Fig. 6D).

Then, we employed the NRCM and NRCF with 500 ng/ml Ga_i_ inhibitor pertussis toxin (PTX) for 2 h before other stimulations. The mRNA expression of Nppa, and Nppb, as well as the ratio of Myh 6/7 showed that PTX pretreatment blocked the attenuating effects of MrgD shRNA on NRCM hypertrophy (Fig. 6E), while it did not enhance the effects of Ad-MrgD on NRCM hypertrophy (Fig. 6F). Morever, the protein expression of α-SMA, TGF-β and collagen I in NRCF showed that PTX pretreatment blocked the antifibrotic effects of MrgD shRNA (Fig. 6G), while it did not enhance the pro-fibrosis effects of Ad-MrgD (Fig. 6H). The similar trends were also seen in the mRNA expressions of Acta2, TGF-β and COL1A1 in NRCF (Figure S12A-S12B).

**Fig. 7:**
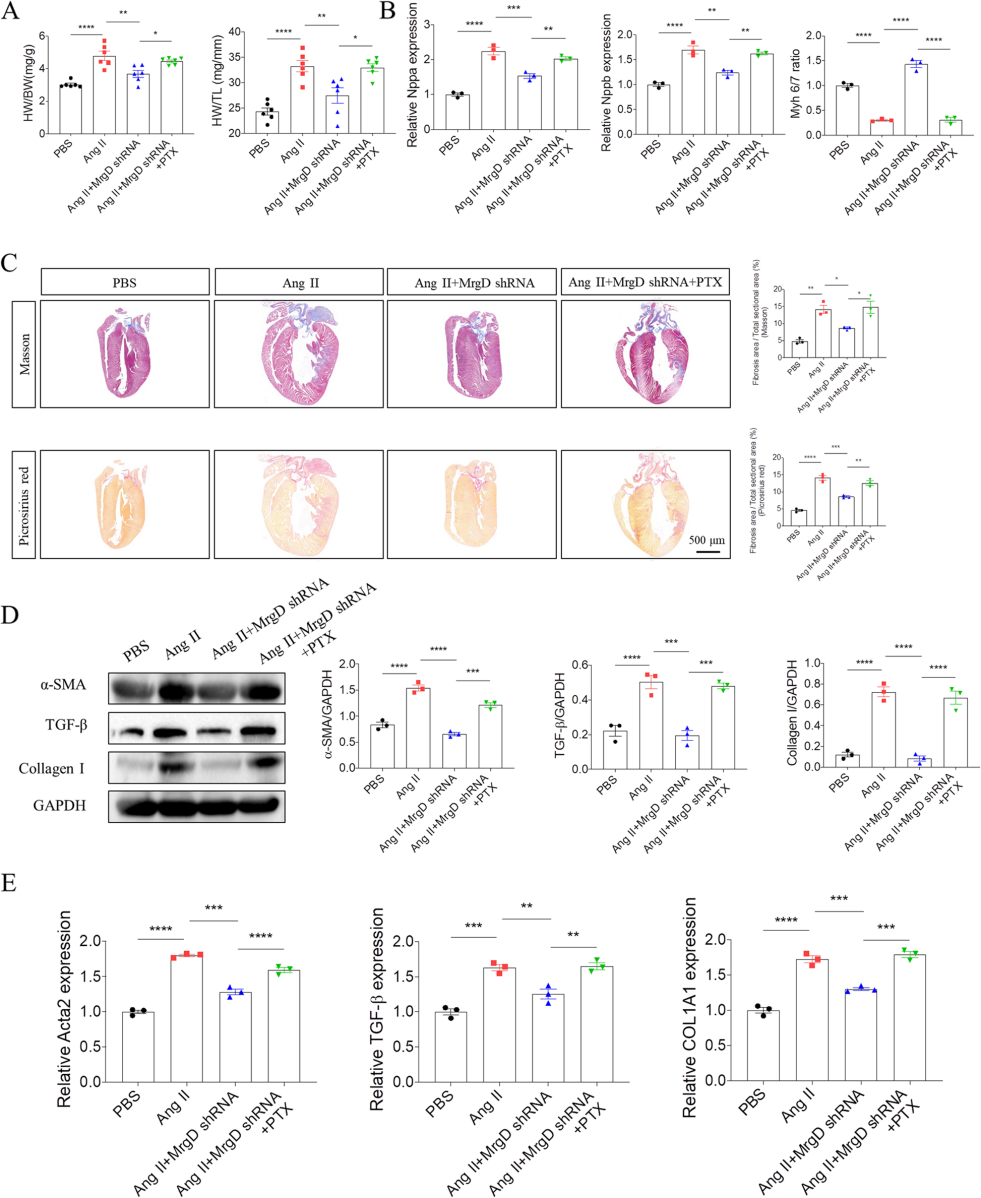
Effects of PTX on the protective role of silencing MrgD in Ang II-induced cardiac remodeling. **A**, HW/BW and HW/TL in the hearts from different groups. **B**, the mRNA levels of Nppa and Nppb, as well as the ratio of Myh 6/7in the hearts from different groups. **C**, PTX pretreatment blocked the protective effects of silencing MrgD against Ang II-induced cardiac fibrosis in rats, as indicated by masson and picrosirius red staining. **D**-**E**, the protein and mRNA levels of α-SMA, TGF-β and collagen I in the hearts from different groups. The results are expressed as mean ± SEM. *n* = 3 biological replicates per group. ^ns^*P* > 0.05, ^*^P < 0.05, ^**^P < 0.01, ^***^P < 0.001, ^****^P < 0.0001

Further, the rats were pretreated with PTX (10 μg/kg [18]) intraperitoneally for 48 h before intra-myocardial injection of MrgD shRNA and 4-week-infusion of Ang II. After harvest, we found that PTX pretreatment hampered the protective effects of silencing MrgD on AngII-induced cardiac dysfunction (Table 4). Besides, the rats in the Ang II + MrgD shRNA + PTX group had a worse cardiac function with increased ratio of HW/TL and HW/BW than those in the Ang II + MrgD shRNA group (Fig. 7A). The similar trend was also seen in the mRNA expression of Nppa, and Nppb, as well as the ratio of Myh 6/7 (Fig. 7B). Also, the Masson and Picrosirius red staining results showed that the rats in the Ang II + MrgD shRNA + PTX group displayed much more collagen deposition in heart tissues than those in the Ang II + MrgD shRNA group (Fig. 7C). In consistent with the histopathological results, we found that PTX impeded the inhibitory effects of silencing MrgD on Ang II-induced increased α-SMA, TGF-β and collagen I protein (Fig. 7D), and Acta2, TGF-β and COL1A1 mRNA (Fig. 7E) expression.

**Table 4:** Either Ala and PD123319 could improve Ang II-induced cardiac dysfunction in the rat

Variables	PBS (*n* = 6)	Ang II (*n* = 6)	Ang II + Ala (*n* = 6)	Ang II + PD123319 (*n* = 6)	Ang II + Ala + PD123319 (*n* = 6)
LVEF (%)	77.55 ± 0.9196	78.95 ± 2.161	76.16 ± 1.916^ ns^	78.21 ± 0.9513^ ns^	79.96 ± 1.846^ ns^
LVFS (%)	47.99 ± 0.8531	49.31 ± 2.203	46.70 ± 1.775^ ns^	48.54 ± 0.9681^ ns^	50.36 ± 1.881^ ns^
LV mass (mg)	1156 ± 44.45	1423 ± 53.10	1194 ± 54.51 ^*^	1190 ± 49.12 ^*^	1119 ± 52.87 ^*^
LVIDs (mm)	4.310 ± 0.1485	3.676 ± 0.1819	4.241 ± 0.2022	4.091 ± 0.1202	3.731 ± 0.1895^ ns^
LVIDd (mm)	8.278 ± 0.1828	7.255 ± 0.2117	7.935 ± 0.1295^ ns^	7.951 ± 0.1818^ ns^	7.511 ± 0.2318^ ns^
LVAW;s (mm)	2.834 ± 0.0642	3.762 ± 0.0573	2.898 ± 0.1117 ^****^	3.359 ± 0.0419 ^**^	3.033 ± 0.0634 ^****^
LVAW;d (mm)	1.764 ± 0.04898	2.410 ± 0.07515	1.872 ± 0.03872 ^****^	1.889 ± 0.02203 ^****^	1.852 ± 0.05904 ^****^
LVPW;s (mm)	2.827 ± 0.08314	3.571 ± 0.09882	3.255 ± 0.03894^ ns^	3.374 ± 0.04458^ ns^	3.296 ± 0.1012^ ns^
LVPW;d (mm)	1.809 ± 0.06843	2.347 ± 0.1110	1.959 ± 1.05446 ^**^	1.930 ± 0.06183 ^**^	2.001 ± 0.04534 ^*^
Heart weight (mg)	1156 ± 40.08	1647 ± 34.69	1222 ± 56.30 ^****^	1261 ± 17.10 ^****^	1200 ± 19.76 ^****^
Body weight (g)	386.3 ± 15.971	358.6 ± 10.37	386.9 ± 14.34^ ns^	402.3 ± 17.10^ ns^	373.0 ± 10.44^ ns^
Tibia length (mm)	48.06 ± 0.2562	48.07 ± 0.2337	47.87 ± 0.2186^ ns^	47.87 ± 0.2186^ ns^	47.78 ± 0.1833^ ns^

Echocardiographic data of mice from different groups. The results are expressed as the mean ± SEM (*LVEF* Left ventricular ejection fraction, *LVFS* Left ventricular fractional shortening, *LVIDs* LV internal diameters at end systole, *LVIDd* LV internal diameters at end diastole, *Volume;s* Volume at end systole, *Volume;d* Volume at end diastole, *LVAW;s* Left ventricular systolic anterior wall, *LVAW;d* Left ventricular diastolic anterior wall, *LVPW;s* Left ventricular systolic posterior wall, *LVPW;d* Left ventricular diastolic posterior wall). (^*^: vers Ang II group)

^ns^*P > *0.05, ^*^P < 0.05, ^**^P < 0.01, ^***^P < 0.001, ^****^P < 0.0001

The above results indicated that silencing MrgD exerts its cardioprotective effects via suppressing Gα_i_ activation.

### Effects of MrgD on signaling pathways

**Fig. 8:**
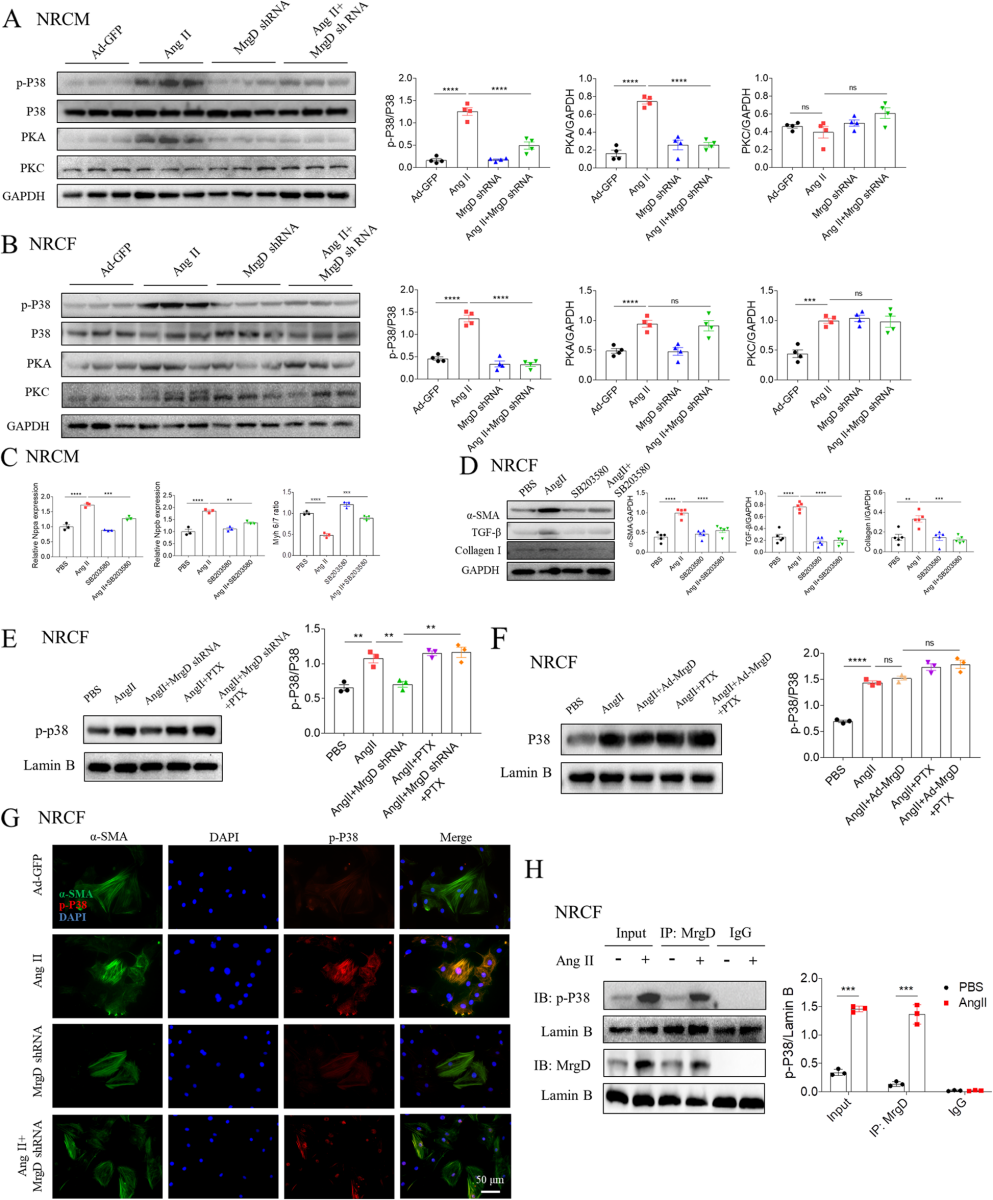
MrgD interacted with p-P38 in the nuclei. **A**, The increase of p-P38 MAPK and PKA induced by Ang II were inhibited by MrgD downregulation in NRCM. **B**, The increase of p-P38 MAPK induced by Ang II was inhibited by MrgD downregulation in NRCF. **C**-**D**, SB203580 pre-treatment ameliorated Ang II-induced NRCM hypertrophy NRCF fibrosis. **E**, MrgD shRNA inhibited Ang II-induced nuclear import of p-P38 in NRCF, which could be reversed by PTX pretreatment. F, Ad-MrgD has no extra effects on enhancing Ang II-induced nuclear import of p-P38 in NRCF. G, immunofluorescence staining of NRCF with antibodies against α-SMA and p-P38 (α-SMA, green; p-P38, red; DAPI, blue; scale bar: 50 μM). H, Co-IP experiments verified that MrgD interacted with p-P38 in the nuclei. The data are expressed as mean ± SEM. *n* = 3, 4 or 5 biological replicates per group. ^ns^*P* > 0.05, ^*^P < 0.05, ^**^P < 0.01, ^***^P < 0.001, ^****^P < 0.0001

Previous studies have shown that Ala exerted its biogical effects via phosphorylation of p38 mitogen-activated protein kinase (p-P38 MAPK) and protein kinase A (PKA) signaling pathways [14, 19, 20]. Hence, we investigated whether these signaling pathways also have a role in this study. The protein expression of p-P38 and PKA were increased in Ang II-treated NRCM, which was inhibited by MrgD knockdown (Fig. 8A). Also, the protein kinase C (PKC) level in NRCM was increased by Ang II treatment; however, MrgD knockdown did not affect this upregulation (Fig. 8A). The level of p-P38 was increased in Ang II-treated NRCF, and which was inhibited by MrgD knockdown (Fig. 8B). However, MrgD knockdown had no effect on the PKA and PKC levels under Ang II stimulation in NRCF (Fig. 8B). Besides, we assessed the activation of CREB, a PKA target, to confirm the activation of PKA signaling in NRCM and NRCF. The results showed that silencing MrgD downregulated Ang II-induced CREB protein expression in NRCMs, but not in NRCFs (Figure S13A-S13B).

As a member of the MAPK pathways, the p38 MAPK signaling cascade was studied in depth to be of fundamental importance in transmitting extracellular signals to the targets or transcription machinery in the cytoplasm and nucleus shortly after stimulation of different environmental stress [21, 22]. We next pre-treated cells with 0.1 µM SB203580, an inhibitor of p38 MAPK, before Ang II induction [23]. The results showed that SB203580 rescued Ang II-induced cardiac hypertrophy (Fig. 8C) and fibrosis (Fig. 8D) in vitro. Then, we found that Ang II or MrgD overexpression induced nuclear import of p-P38, which could be reversed by silencing MrgD. PTX pretreatment blocked the protective effects of MrgD shRNA against Ang II-stimulated accumulation of p-P38 in the nuclear (Fig. 8E-F). The immunostaining in NRCFs supported that MrgD knockdown inhibited nuclear translocation of p-P38 under Ang II stimulation (Fig. 8G). Further, the Co-IP experiments performed in nuclear protein of NRCMs showed MrgD coimmunoprecipitated with p-P38, indicating that MrgD interacted with p-P38 in the nucleus (Fig. 8H).

## Discussion

Ala, an identified component of RAS, exerts a protective effect on Ang II-induced cardiac hypertrophy via its receptor MrgD [14]. Ala/MrgD axis is reported to have a counter-regulatory effect against Ang II/AT1R axis following Ang-(1–7)/Mas axis. However, in the present study, we found that MrgD overexpression resulted in NRCM hypertrophy and NRCF fibrosis. Ala treatment significantly attenuated the Ang II-induced cardiac hypertrophy and fibrosis via inhibition of the downstream signaling pathway.

There are three axes of RAS, including Ang II/AT1R, Ang-(1–7)/Mas, and Ala/MrgD. Previous studies have also demonstrated that multiple novel biological members such as Ang-(1–7)/Mas and Ala/MrgD counteract the fibrogenic and proliferative effects exerted by those in the ACE/Ang II/AT1R axis [14, 24]. Here, we found that MrgD and its ligand differentially regulate cardiac hypertrophy and fibrosis, and Ala may be an antagonist that combines with its receptor MrgD for the downstream actions. Also, in our study, MrgD KO mice was induced by intraperitoneal injection of tamoxifen in Cre + /–MrgDflox/flox mice. As known, the target gene can’t be knocked out completely by tamoxifen. Therefore, MrgD is an incomplete knockout in this status.

Interestingly, Ala increased MrgD expression in NRCM and NRCF, so did Ang II. A previous study showed that losartan treatment upregulated the level of AT1R in the left ventricle of rats [25]. Since losartan is a competitive AT1R blocker, in vivo chronic administration resulted in an increase in AT1R abundance in the heart in a negative feedback manner [25]. The current results indicated that Ala, as a ligand of MrgD, is a MrgD blocker that elevates MrgD expression in a negative feedback manner. Besides, we found that either Ala or PD123319 (another MrgD receptor antagonist) could ameliorate pro-hypertrophic and pro-fibrotic effects of MrgD overexpression in vitro and in vivo, while use of these two agents (Ala and PD123319 together) have no further effects, which may further enhance our conclusion.

Notably, the expression of the MrgD was mainly detected in the cell membrane and nuclear region, as described previously [26]. Further, Ala administration attenuated Ang II-induced increase in MrgD levels in nucleus. Also, Ala improved cardiac hypertrophy and fibrosis in MrgD overexpression cells, supporting its role as a antagonist rather than agonist of MrgD receptor. These results demonstrated that Ala improved cardiac hypertrophy and fibrosis via attenuating the nuclear import of MrgD.

The previous study reported a dilated cardiomyopathy phenotype observed in the MrgD knockout mice [26], which seemed to be inconsistent with our central findings. However, different studies may draw completely different conclusions by using different experimental animals (including rat, mouse, etc.) at different developmental stages. Especially, heart development is a complex and dynamic process [27]. Unlike the embryonic stage, the neonatal hearts of mice and rats appear to reach full histological maturation after birth [28]. Thus, the previous could only emphasize the importance of MrgD in myocardial development [26], but could not be over-extended to explain its role in the changes of cardiac function after the heart has matured. Since cardiac hypertrophy and fibrosis are considered as the adult diseases, we conducted experiments in the adult rat or mice, and our results supported that MrgD could be a therapeutic target in the future. It's worth noting that drugs targeting MrgD may contraindicated in pregnant women due to their teratogenic effects.

As a member of the GPCR family associated with crucial physiological signaling pathways involved in organ development and function [29], MrgD was initially found to regulate pain pathways [30, 31]. Ala is a heptapeptide that performs many functions by interacting with MrgD [32]. In canonical GPCR signaling at the cell membrane, ligand binding to a receptor results in a change in receptor conformation and causes the activation of G proteins, a GTP-binding protein [33]. Gαi-coupled receptors has been reported to play an vital role in protecting the heart from injurys [34]. In our study, we found that Ala reversed Ang II-induced downregulation of Gα_i._ MrgD knockdown has the same effects as Ala, while MrgD overexpression reduced the levels of Gα_i1_, Gα_i2_ and Gα_i3_. Besides, pre-treatment of Gαi inhibition PTX hampered the antifibrotic effects of MrgD knockdown in vitro and in vivo, which may support that Ala or MrgD shRNA exerts their cardioprotective effects via activating Gαi signaling pathway under Ang II stimulation.

The cellular response of p38 to environmental stresses appeared as the phosphorylation-dependent nuclear translocation of cytosolic p38 [35, 36], which enabled it to initiate or co-regulate the nuclear translocation or other biological processes of other functional proteins in the cytosol [21, 37, 38]. Though the exact mechanisms underlying the nuclear translocation of p38 were still not fully understood, the use of nuclear translocation inhibitors of p38 or other members in MAPK pathways has been currently reported to be novel therapeutic targets for inflammatory or other various diseases [39, 40]. Also, the dysregulation of p38 was believed to be closely related to the different pathological changes of cardiovascular system [22, 41]. Under the premise that the downstream signaling pathway, such as cAMP-PKA is inhibited if the ligand interacts with Gα_i_ [42, 43], we found that MrgD downregulation only attenuated the increase of p-P38 but not PKC and PKA in Ang II-induced both NRCF and NRCMs, so as to alleviate NRCM hypertrophy and ameliorate NRCF fibrosis via MrgD receptor interacting with Gα_i_. Also, silencing MrgD downregulated Ang II-induced activation of a PKA target, CREB, in NRCMs, but not in NRCFs, which further suggesting that PKA signaling may not be involved in the biological effects of MrgD mentioned in our study.

Thus, the Co-IP was applied to examine the interaction between MrgD and p-P38, and the results suggested that MrgD could bind to p-P38 and promote its entry into the nucleus, thereby initiating downstream functional signaling pathways under Ang II stimulation.

Currently, standard or conventional medications for chronic heart failure include diuretics, angiotensin converting enzyme inhibitors (ACEI), Ang II receptor antagonists [32], β-blockers, and aldosterone receptor antagonists [44]. However, other than that, there has been no breakthrough in the research of new drugs. Heart failure mortality and re-hospitalization rates remain high [45], which imposes a huge financial burden on society and patients. Therefore, it is urgent to find new therapeutic targets for heart failure. Here, we found that MrgD-mediated signal transduction was closely related to the pathophysiological process of cardiac remodeling. At the same time, silencing MrgD can prevent the occurrence and development of heart failure, indicating that MrgD may be a promising therapeutic target.

## Conclusions

The current study sheds light on the protective role of silencing MrgD expression in alleviating AngII-induced cardiac hypertrophy and fibrosis. Ala, as an inhibitory ligand of MrgD, attenuates Ang II-induced cardiac remodeling. These results paved a way to develop novel therapeutic agents to inhibit MrgD in clinical practice and improve cardiac remodeling in the future.

## Supplementary Information

**Additional file 1:** **Table S1.** List of utilized primers for qRT-PCR.

**Additional file 2:** **Figure S1.** Expression of MrgD in different pathological models.

**Additional file 3:** **Figure S2.** The transfection efficiency of Ad-MrgD, and MrgD shRNA in NRCM and NRCF.

**Additional file 4:** **Figure S3.** Effects of MrgD on NRCF fibrosis.

**Additional file 5:** **Figure S4.** The MrgD expression in the heart after Ad-MrgD microinjection into the LV.

**Additional file 6: Figure S5.** The MrgD expression in the heart of MrgD KO mice.

**Additional file 7:** **Figure S6.** Effects of Ala on NRCM hypertrophy and NRCF fibrosis.

**Additional file 8:** **Figure S7.** Ala attenuated Ad-MrgD-induced NRCM hypertrophy and NRCF fibrosis.

**Additional file 9:** **Figure S8.** Effects of Ala or PD123319 on Ang II-induced cardiac hypertrophy and fibrosis.

**Additional file 10:** **Figure S9.** Effects of Ala-MrgD on AC16 hypertrophy.

**Additional file 11:** **Figure S10.** Effects of Ala on Ad-MrgD-induced cAMP levels.

**Additional file 12:** **Figure S11.** Effects of Ala on Gα_i_ in NRCM and NRCF.

**Additional file 13:** **Figure S12.** Ga_i_ inhibitor reversed the effect of MrgD knockdown in NRCF.

**Additional file 14:** **Figure S13.** Effects of MrgD knockdown on Ang II-induced CREB protein expression. A-B, Effects of MrgD knockdown on Ang II-induced CREB protein expression in NRCM (A) or NRCF (B). The results are expressed as mean ± SEM. *n*=3 biological replicates per group. ^ns^*P* > 0.05, *P < 0.05, **P < 0.01, ***P < 0.001, ****P < 0.0001.

## Data Availability

Not applicable.

## Abbreviations

Ala: Alamandine
Ang: Angiotensin
GPCR: G protein-coupled receptor
MrgD: Mas-related G protein-coupled receptor, member D
NRCF: Neonatal rat cardiac fibroblasts
NRCM: Neonatal rat cardiomyocytes
RAAS: Renin–angiotensin–aldosterone system
CVDs: Cardiovascular diseases
AT1R: Angiotensin type 1 receptor
KO: Knockout
α-SMA: Alpha-smooth muscle actin
TGF-β: Transforming growth factor-beta
PFA: Paraformaldehyde
HE: Hematoxylin–eosin
WGA: Wheat germ agglutinin
Co-IP: Coimmunoprecipitation
MAPK: Mitogen-activated protein kinase
PKA: Protein kinase A
