# Supplementary material for: Nuclear import of Mas-related G protein-coupled receptor member D induces pathological cardiac remodeling
Source: Cell Commun Signal. 2023 Jul 24;21:181. doi: 10.1186/s12964-023-01168-3 (PMC10364433; doi:10.1186/s12964-023-01168-3)
Supplement: Supplementary file 2 — Additional file 1: Table S1. List of utilized primers for qRT-PCR. [file 12964_2023_1168_MOESM1_ESM.doc]

Table S1. List of utilized primers for qRT-PCR.

| Gene | Species | Forward primer | Reverse primer |
| --- | --- | --- | --- |
| Nppa | Rat | GAGCAAATCCCGTATACAGTGC | ATCTTCTACCGGCATCTCCTCC |
| Nppb | Rat | GCTGCTGGAGCTGATAAGAGAA | GTTCTTTTGTAGGGCCTTGGTC |
| Myh6 | Rat | ATCTGATGGATTTCAAGAACC | CTCTGAGACGGGTTGACTTC |
| Myh7 | Rat | ACAATCCACGATGCAGAAGCT | GGGCCTTGGTCCTTTGAGA |
| Acta2 | Rat | GCATCCACGAAACCACCTA | CACGAGTAACAAATCAAAGC |
| TGF-β | Rat | TCTGCATTGCACTTATGCTGA | AAAGGGCGATCTAGTGATGGA |
| COL1A1 | Rat | GCTCCTCTTAGGGGCCACT | CCACGTCTCACCATTGGGG |
| MrgD | Rat | CACTGGCCCTCCTGATGAA | GGATGCCAGAATTGAACACAGA |
| GAPDH | Rat | GGCACAGTCAAGGCTGAGAATG | ATGGTGGTGAAGACGCCAGTA |
| MrgD | Mice | TTTTCAGTGACATTCCTCGCC | GCACATAGACACAGAAGGGAGA |
| GAPDH | Mice | AGGTCGGTGTGAACGGATTTG | TGTAGACCATGTAGTTGAGGTCA |
