# Supplementary figures and images for: Nuclear import of Mas-related G protein-coupled receptor member D induces pathological cardiac remodeling
Source: Cell Commun Signal. 2023 Jul 24;21:181. doi: 10.1186/s12964-023-01168-3 (PMC10364433; doi:10.1186/s12964-023-01168-3)

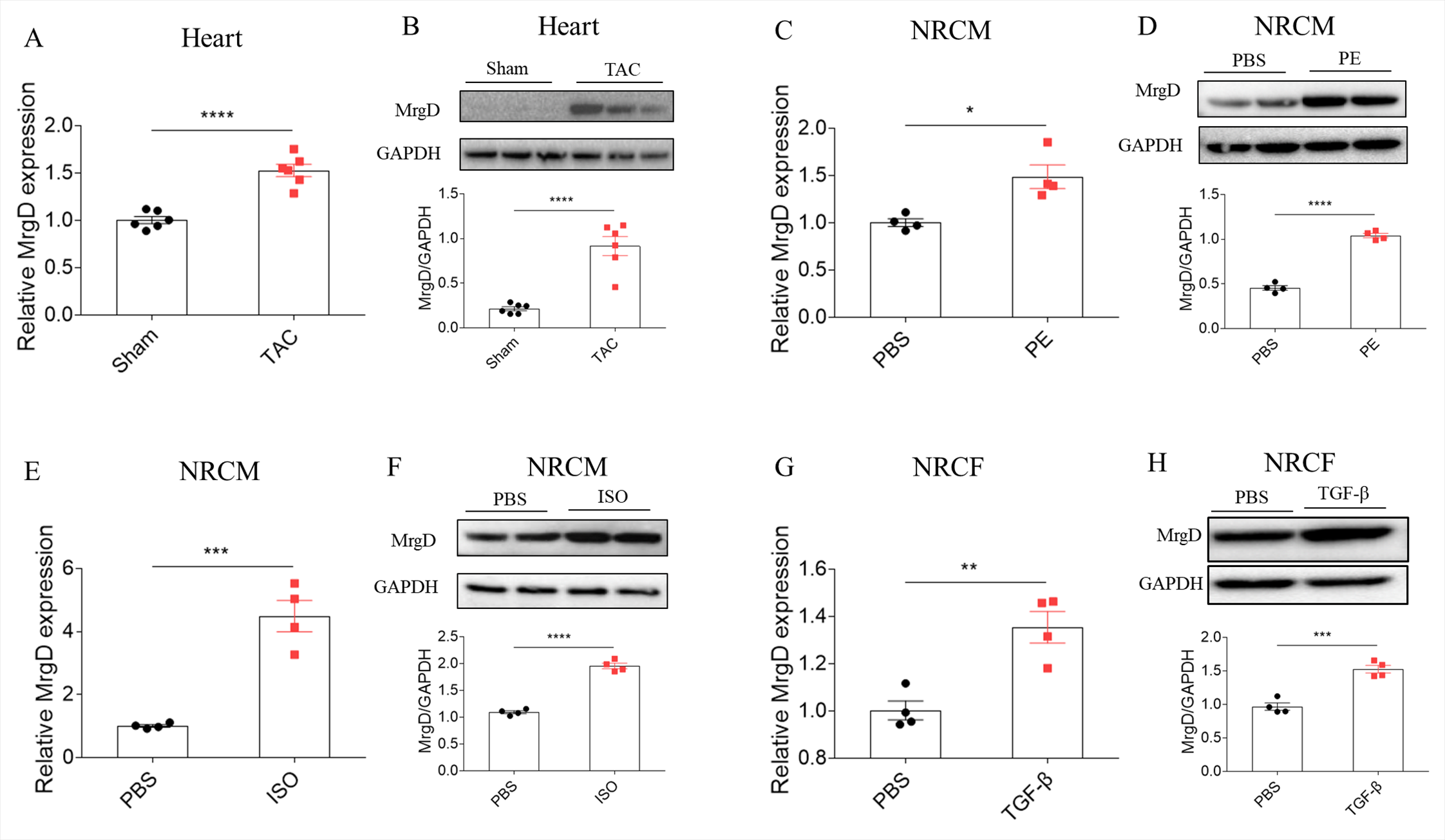

Supplement: Supplementary file 3 — Additional file 2: Figure S1. Expression of MrgD in different pathological models. [file 12964_2023_1168_MOESM2_ESM.tif]

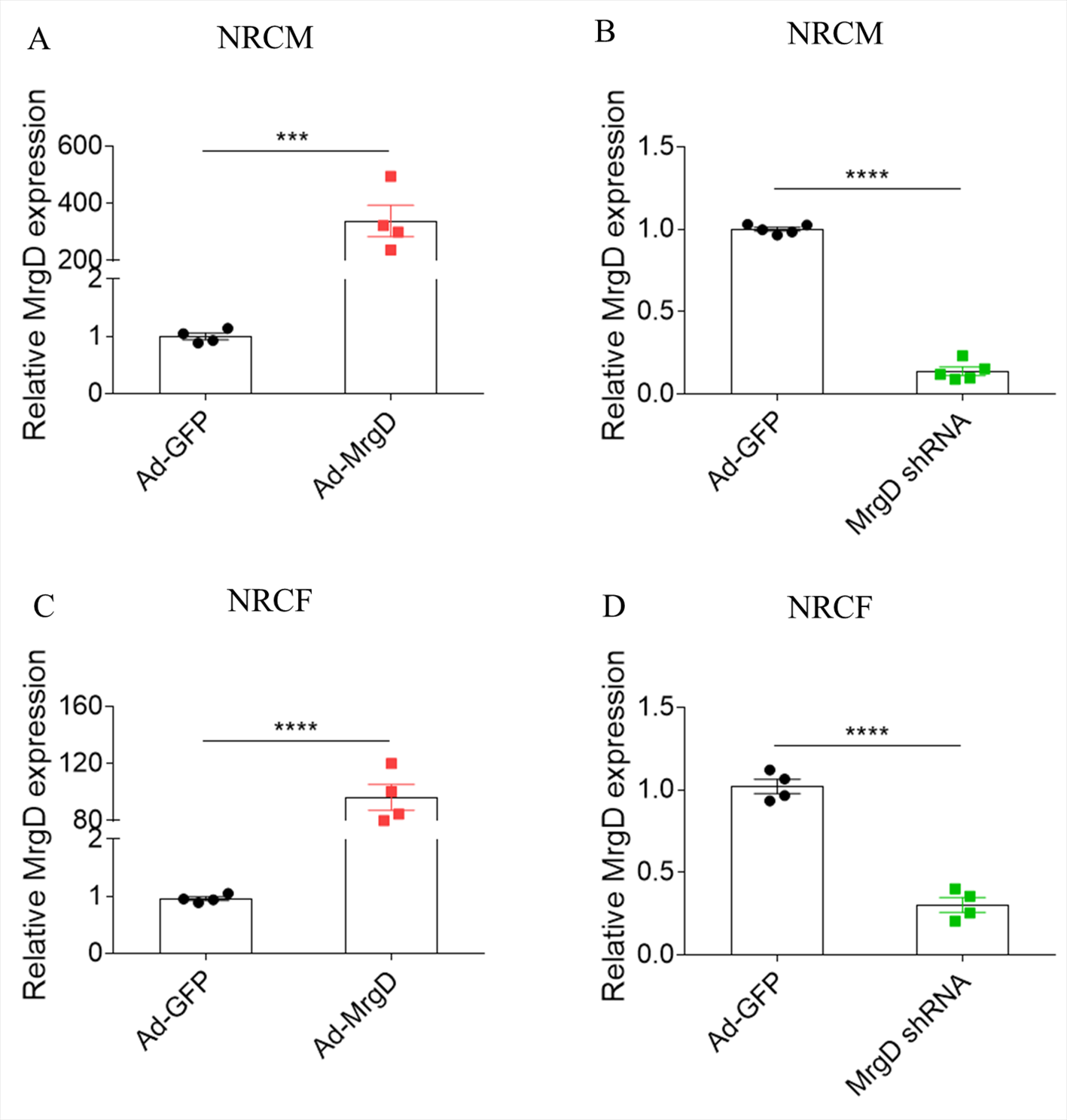

Supplement: Supplementary file 4 — Additional file 3: Figure S2. The transfection efficiency of Ad-MrgD, and MrgD shRNA in NRCM and NRCF. [file 12964_2023_1168_MOESM3_ESM.tif]

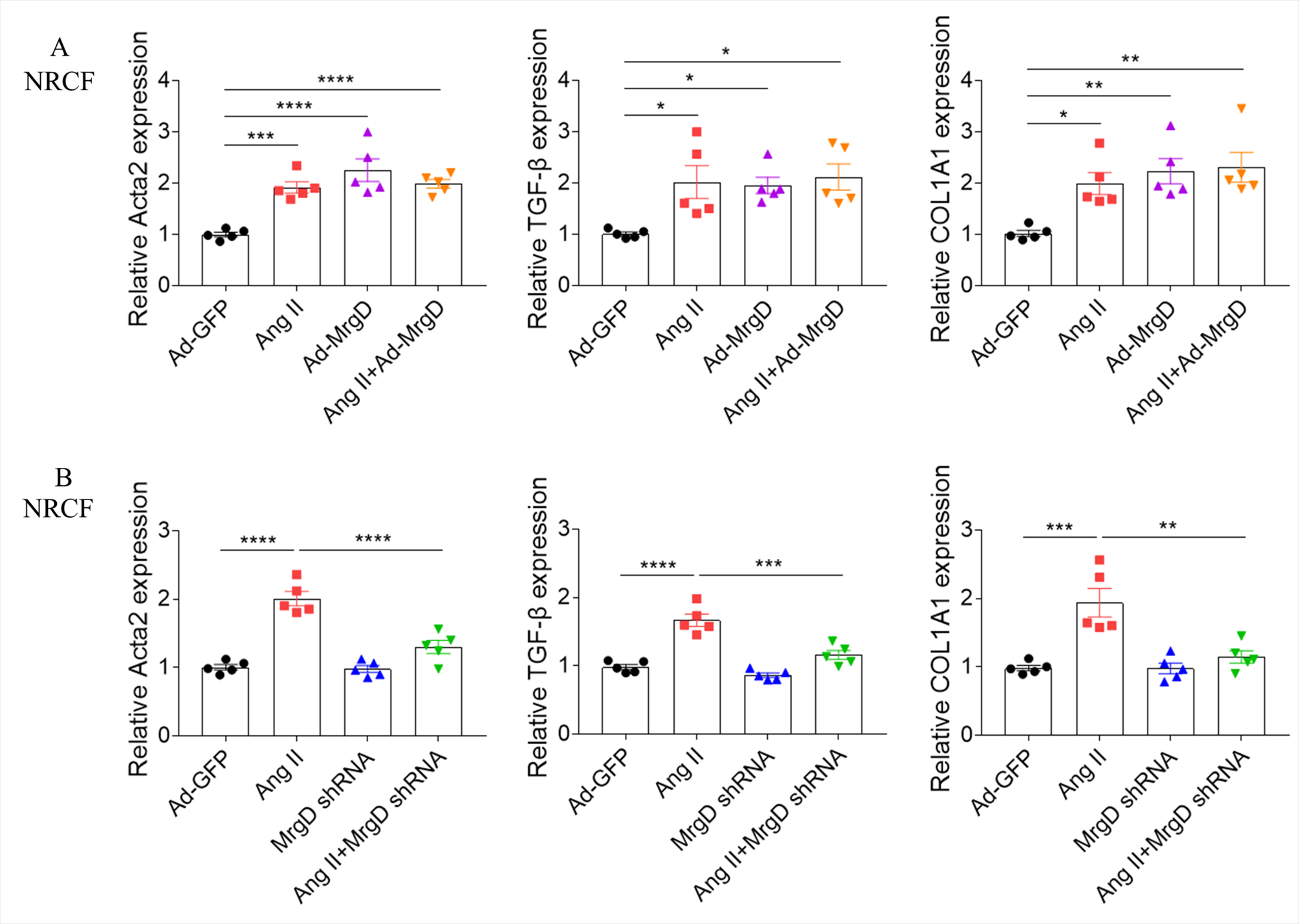

Supplement: Supplementary file 5 — Additional file 4: Figure S3. Effects of MrgD on NRCF fibrosis. [file 12964_2023_1168_MOESM4_ESM.tif]

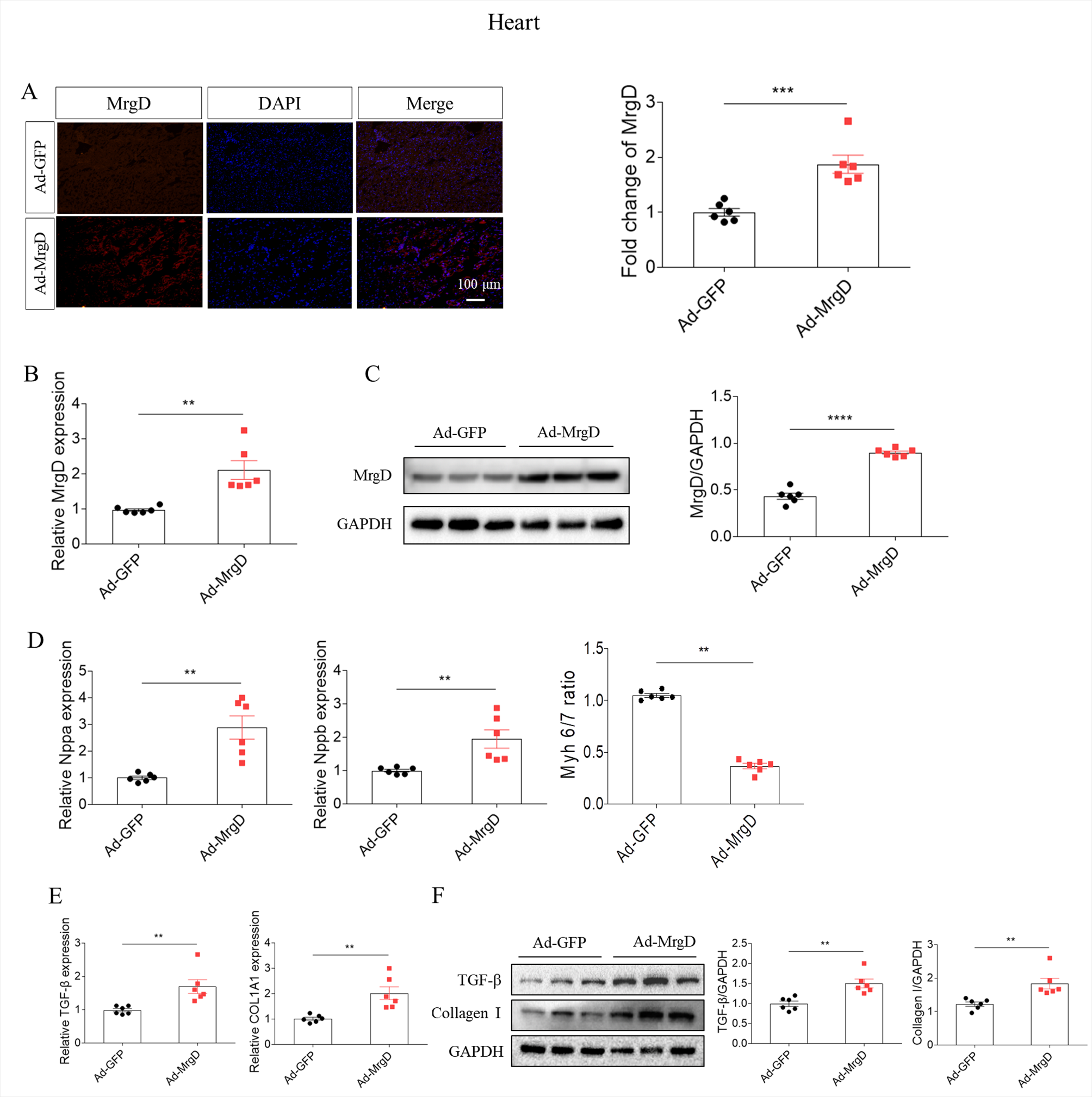

Supplement: Supplementary file 6 — Additional file 5: Figure S4. The MrgD expression in the heart after Ad-MrgD microinjection into the LV. [file 12964_2023_1168_MOESM5_ESM.tif]

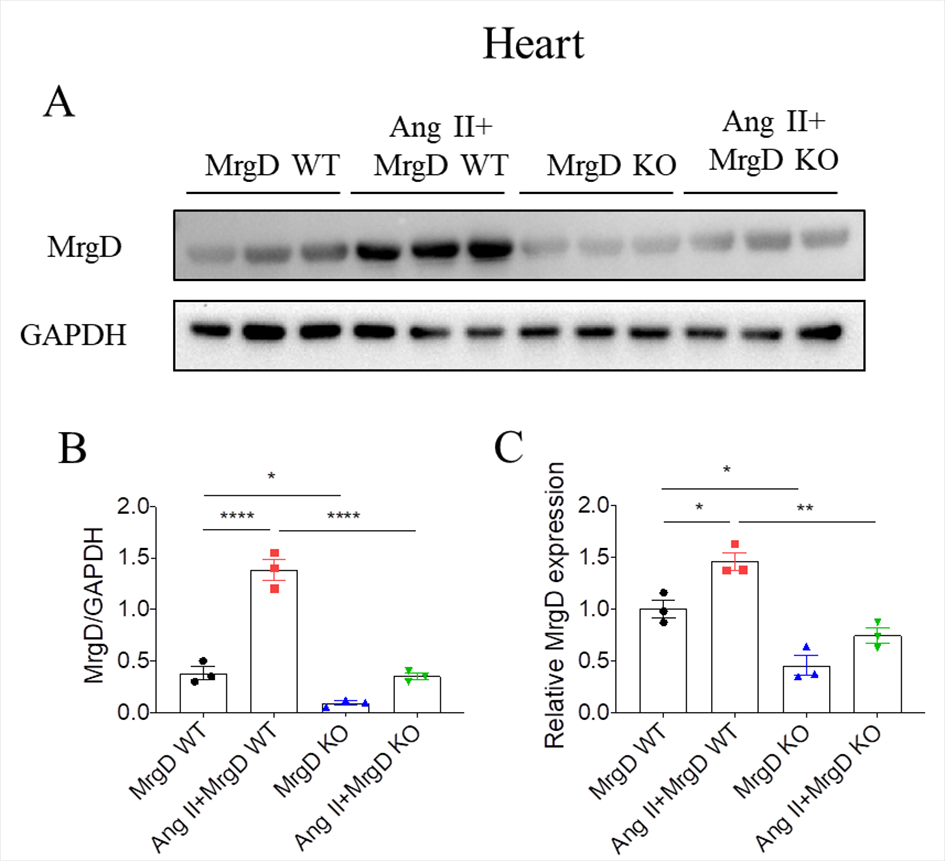

Supplement: Supplementary file 7 — Additional file 6: Figure S5. The MrgD expression in the heart of MrgD KO mice. [file 12964_2023_1168_MOESM6_ESM.tif]

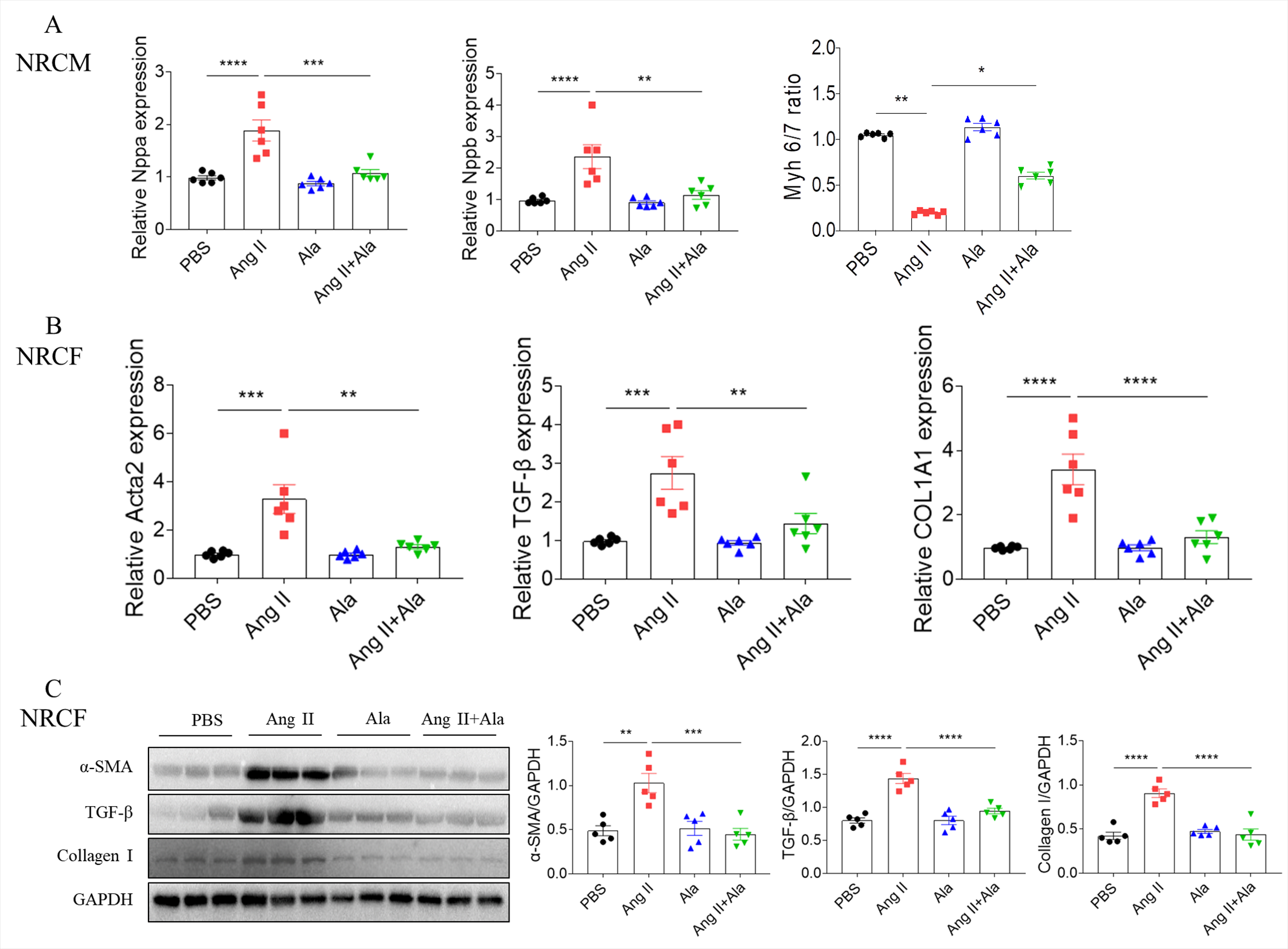

Supplement: Supplementary file 8 — Additional file 7: Figure S6. Effects of Ala on NRCM hypertrophy and NRCF fibrosis. [file 12964_2023_1168_MOESM7_ESM.tif]

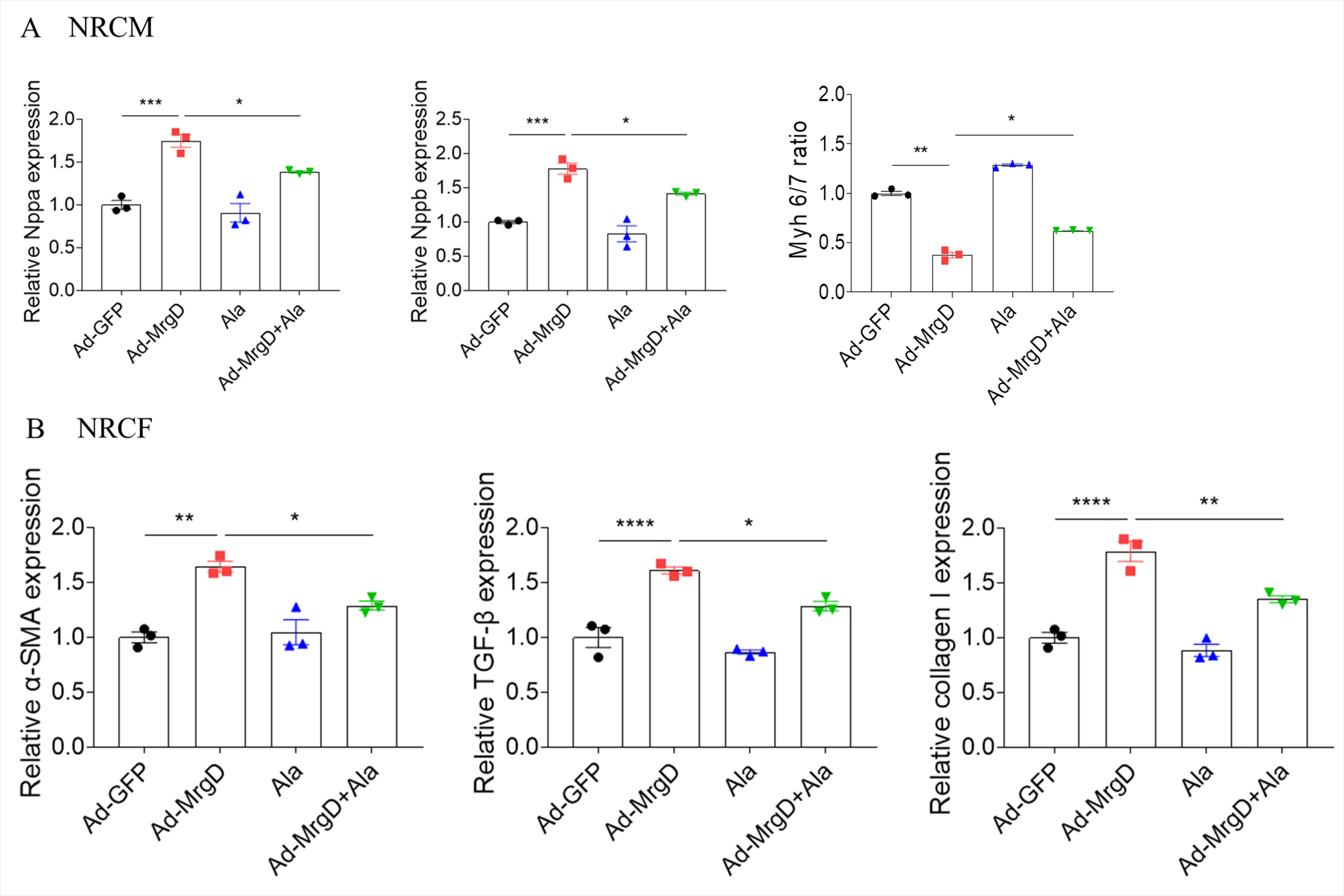

Supplement: Supplementary file 9 — Additional file 8: Figure S7. Ala attenuated Ad-MrgD-induced NRCM hypertrophy and NRCF fibrosis. [file 12964_2023_1168_MOESM8_ESM.tif]

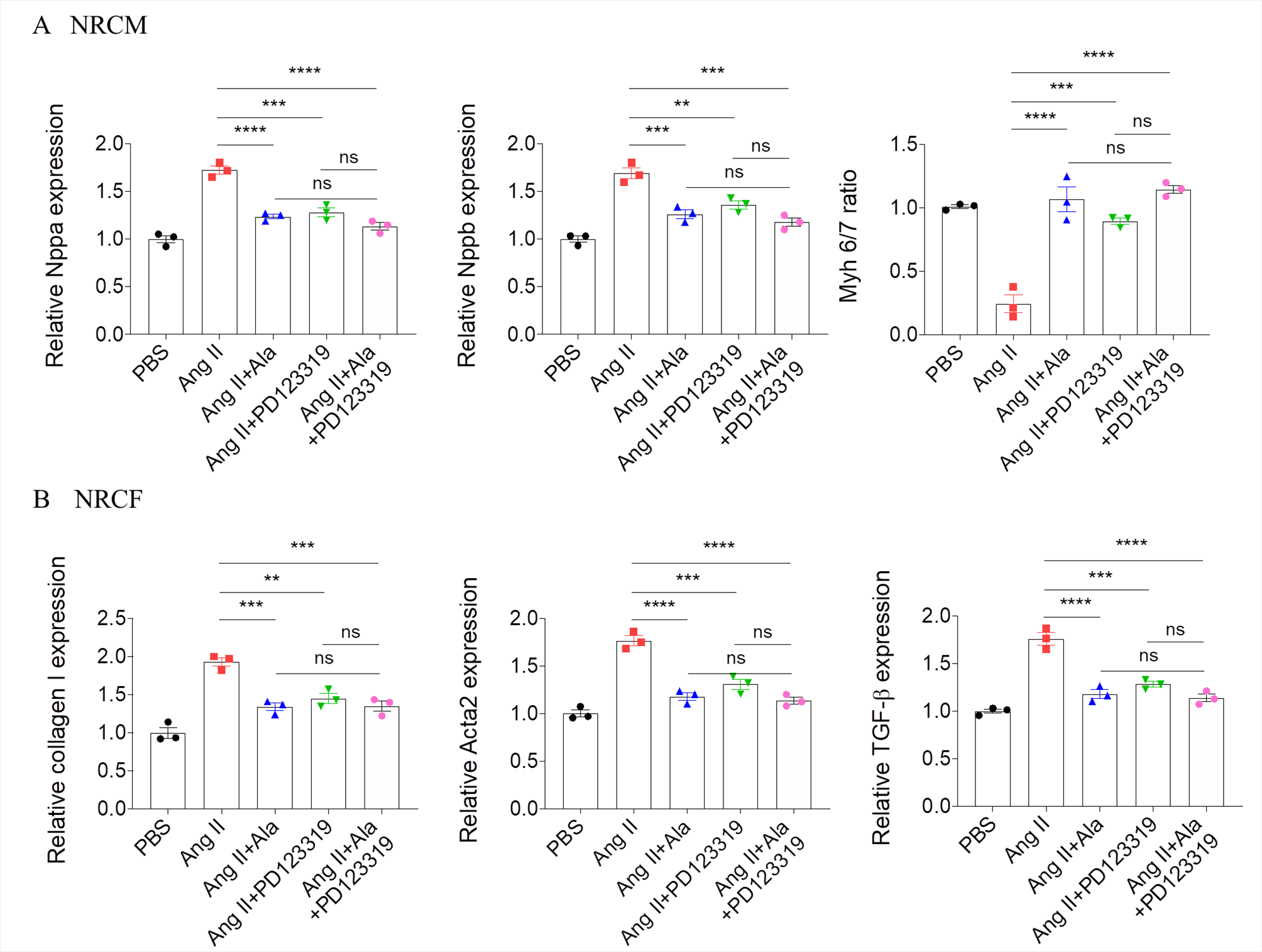

Supplement: Supplementary file 10 — Additional file 9: Figure S8. Effects of Ala or PD123319 on Ang II-induced cardiac hypertrophy and fibrosis. [file 12964_2023_1168_MOESM9_ESM.tif]

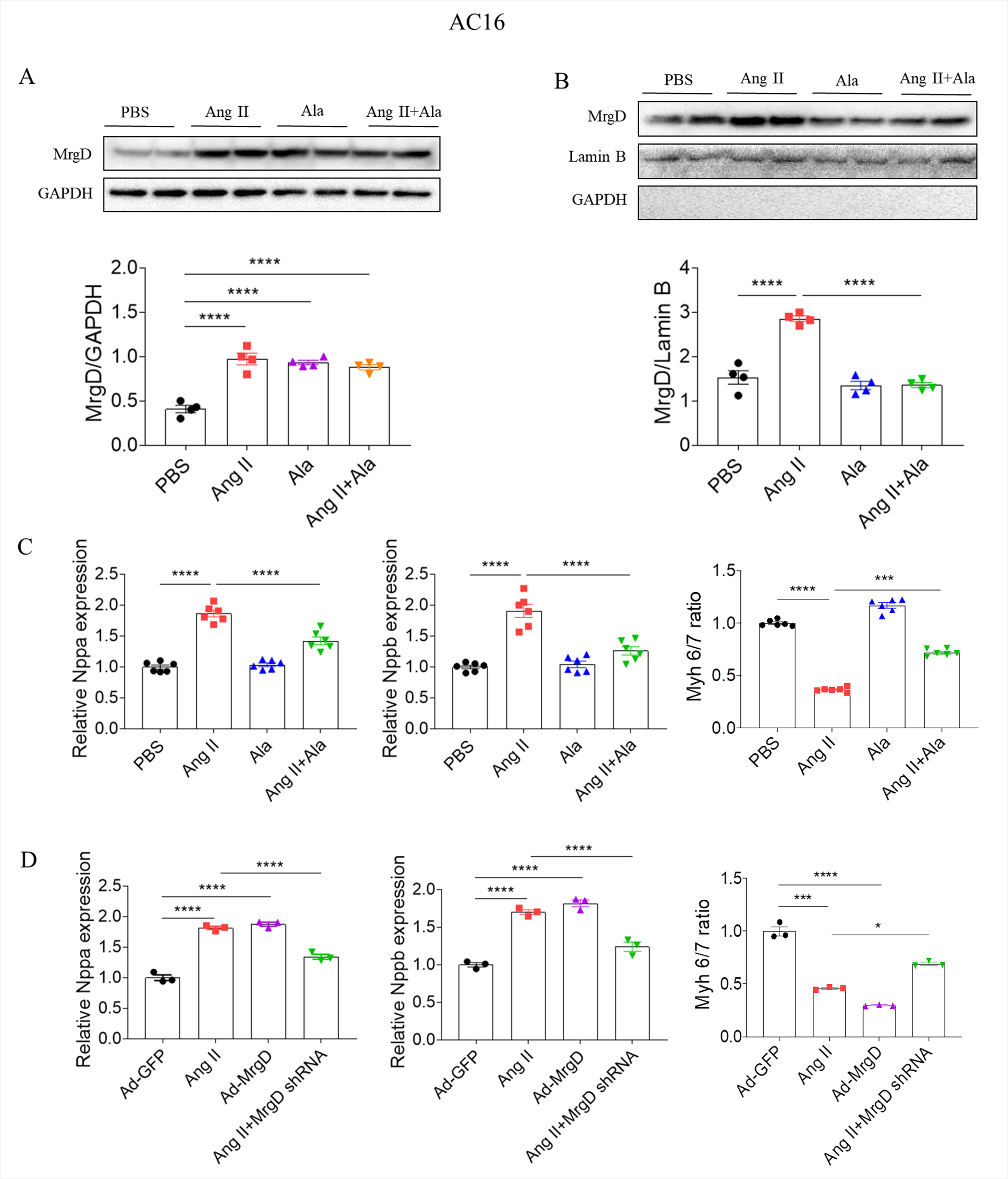

Supplement: Supplementary file 11 — Additional file 10: Figure S9. Effects of Ala-MrgD on AC16 hypertrophy. [file 12964_2023_1168_MOESM10_ESM.tif]

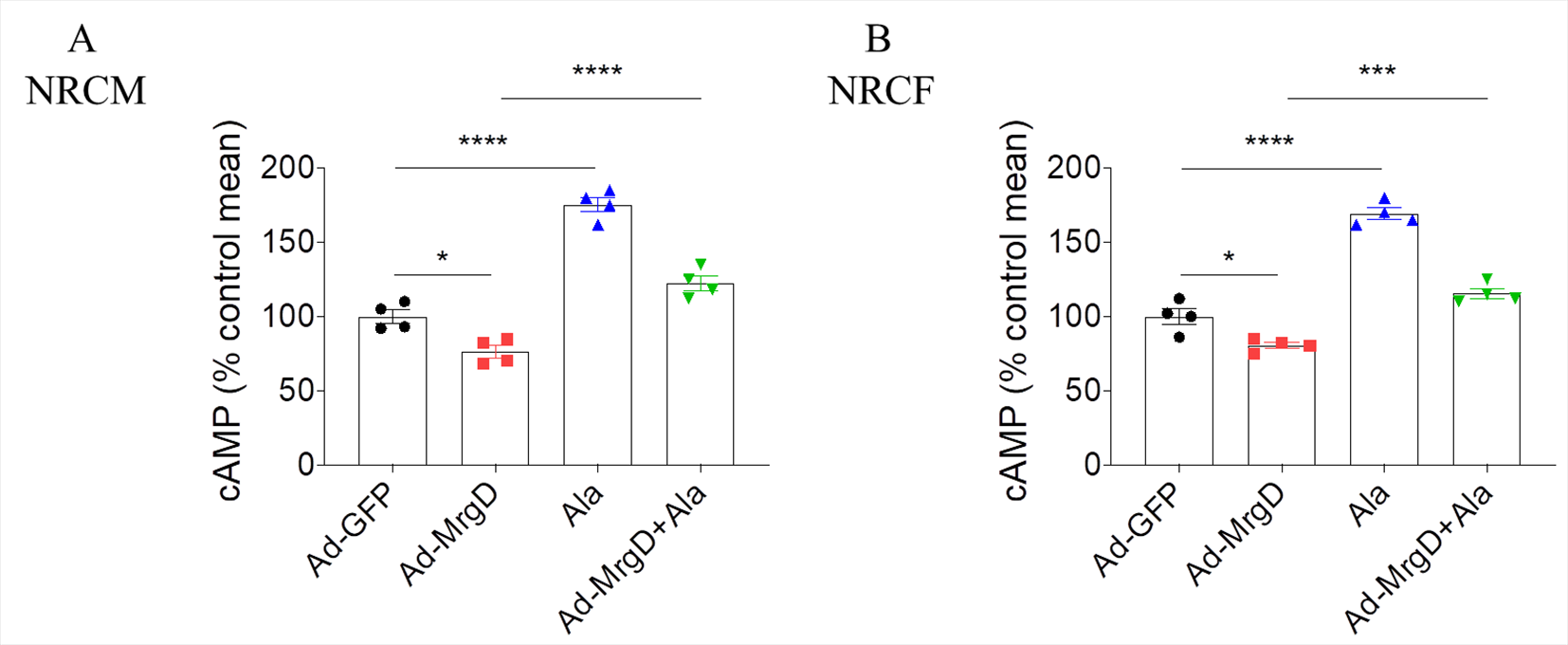

Supplement: Supplementary file 12 — Additional file 11: Figure S10. Effects of Ala on Ad-MrgD-induced cAMP levels. [file 12964_2023_1168_MOESM11_ESM.tif]

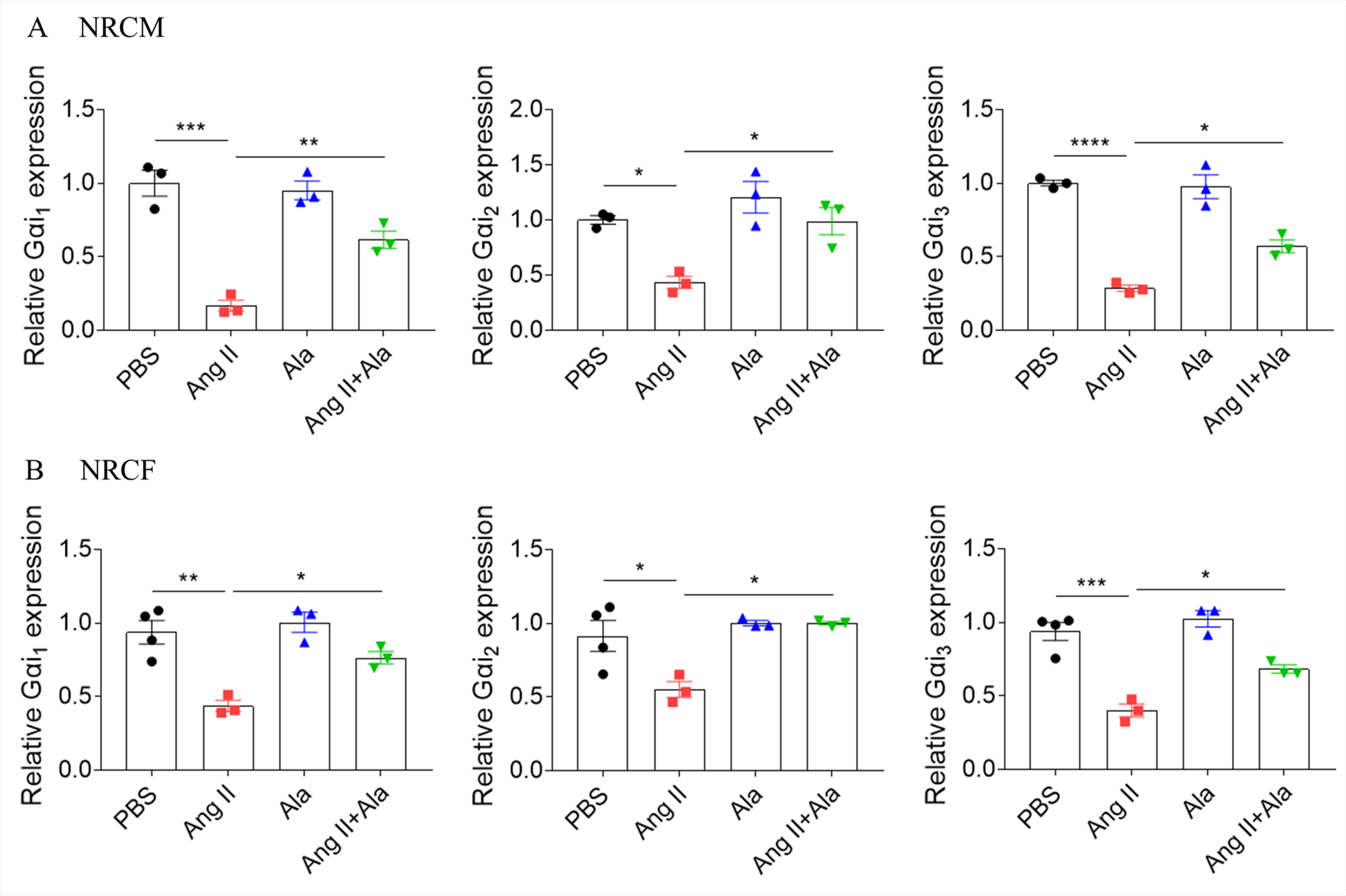

Supplement: Supplementary file 13 — Additional file 12: Figure S11. Effects of Ala on Gαi in NRCM and NRCF. [file 12964_2023_1168_MOESM12_ESM.tif]

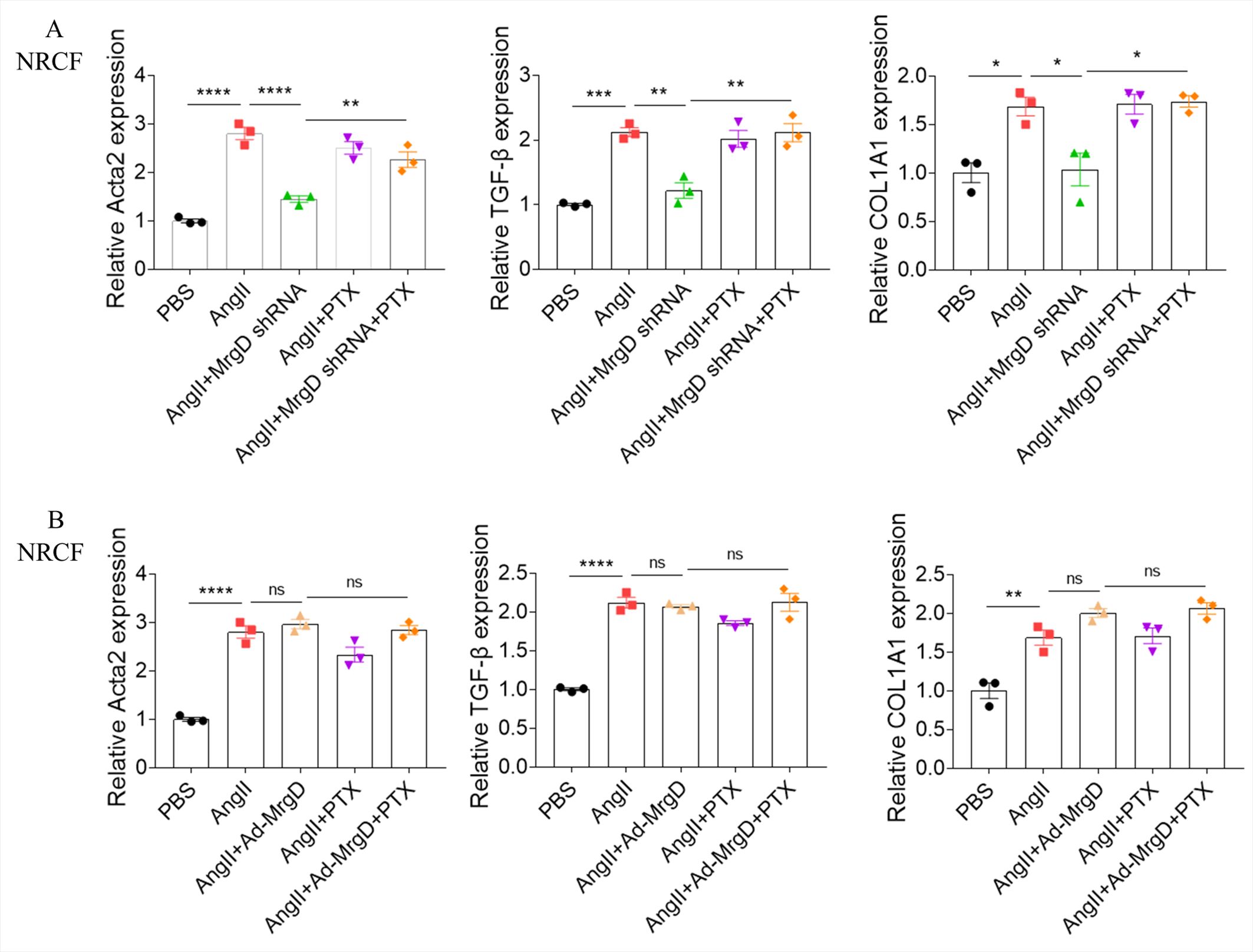

Supplement: Supplementary file 14 — Additional file 13: Figure S12. Gai inhibitor reversed the effect of MrgD knockdown in NRCF. [file 12964_2023_1168_MOESM13_ESM.tif]

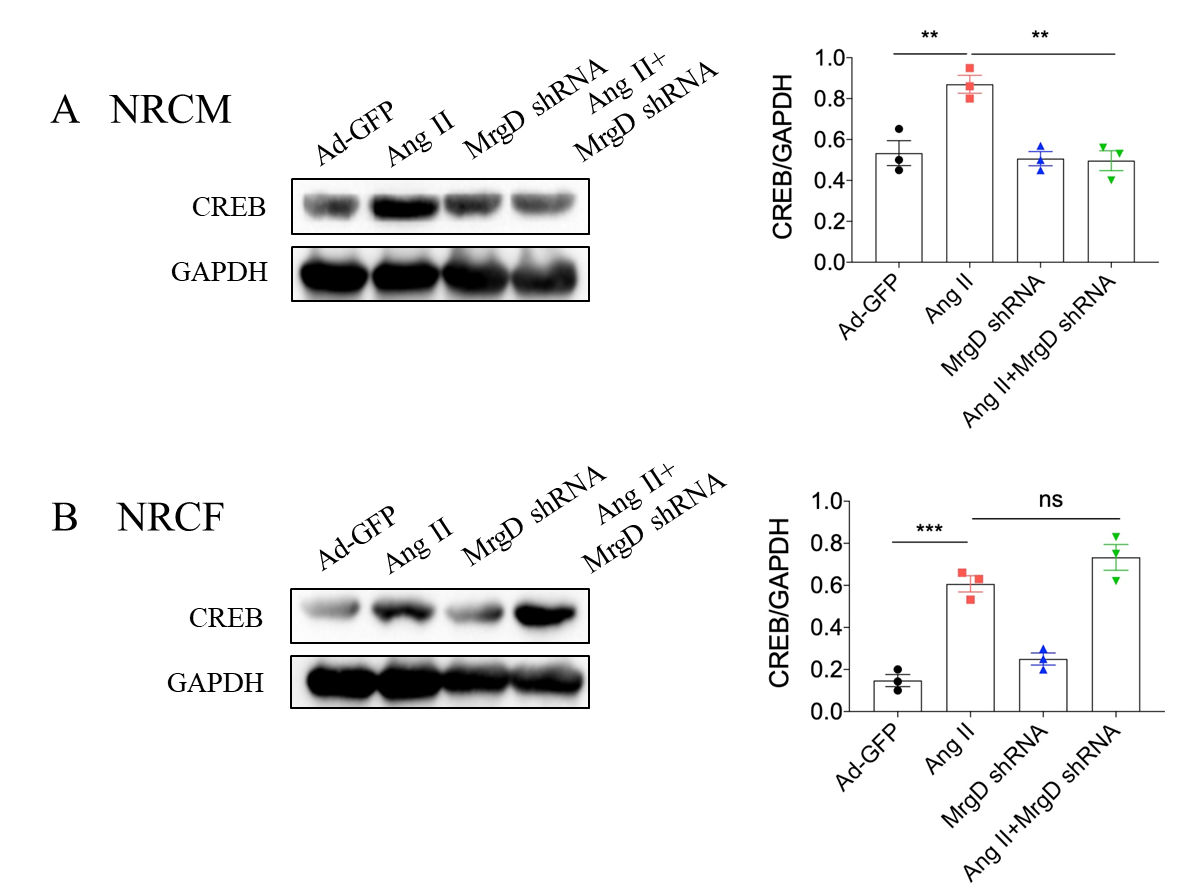

Supplement: Supplementary file 15 — Additional file 14: Figure S13. Effects of MrgD knockdown on Ang II-induced CREB protein expression. A-B, Effects of MrgD knockdown on Ang II-induced CREB protein expression in NRCM (A) or NRCF (B). The results are expressed as mean ± SEM. n=3 biological replicates per group. nsP > 0.05, *P < 0.05, **P < 0.01, ***P < 0.001, ****P < 0.0001. [file 12964_2023_1168_MOESM14_ESM.tif]
